# Homodimeric complexes of the 90–231 human prion: a multilayered computational study based on FMO/GRID-DRY approach

**DOI:** 10.1007/s00894-022-05244-2

**Published:** 2022-08-02

**Authors:** Roberto Paciotti, Loriano Storchi, Alessandro Marrone

**Affiliations:** 1 Department of Pharmacy, University “G d’Annunzio” of Chieti-Pescara, Chieti, Italy; 2 Molecular Discovery Limited, Middlesex, London, UK

**Keywords:** Prion, E200K, Protein–protein interaction, Homodimeric complex, FMO, GRID-DRY, MEP, ATOMIF

## Abstract

The molecular interaction properties and aggregation capabilities disclosed by PrP-E200K, a pathogenic mutant of the human prion protein, were investigated in detail using multilayered computational approaches. In a previous work, we reported that the electrostatic complementarity between region1 (negative) and region3 (positive) has been assumed to lead to a head-to tail interaction between 120 and 231 PrP-E200K units and to initiation of the aggregation process. In this work, we extended the PrP-E200K structure by including the unstructured 90–120 segment which was found to assume different conformations. Plausible models of 90–231 PrP-E200K dimers were calculated and analyzed in depth to identify the nature of the involved protein–protein interactions. The unstructured 90–120 segment was found to extend the positively charged region3 involved in the association of PrP-E200K units which resulted to be driven by hydrophobic interactions. The combination of molecular dynamics, protein–protein docking, grid-based mapping, and fragment molecular orbital approaches allowed us to provide a plausible mechanism of the early state of 90–231 PrP-E200K aggregation, considered a preliminary step of amyloid conversion.

**Supplementary Information:**

The online version contains supplementary material available at 10.1007/s00894-022-05244-2.

## Introduction

**Fig. 1:**
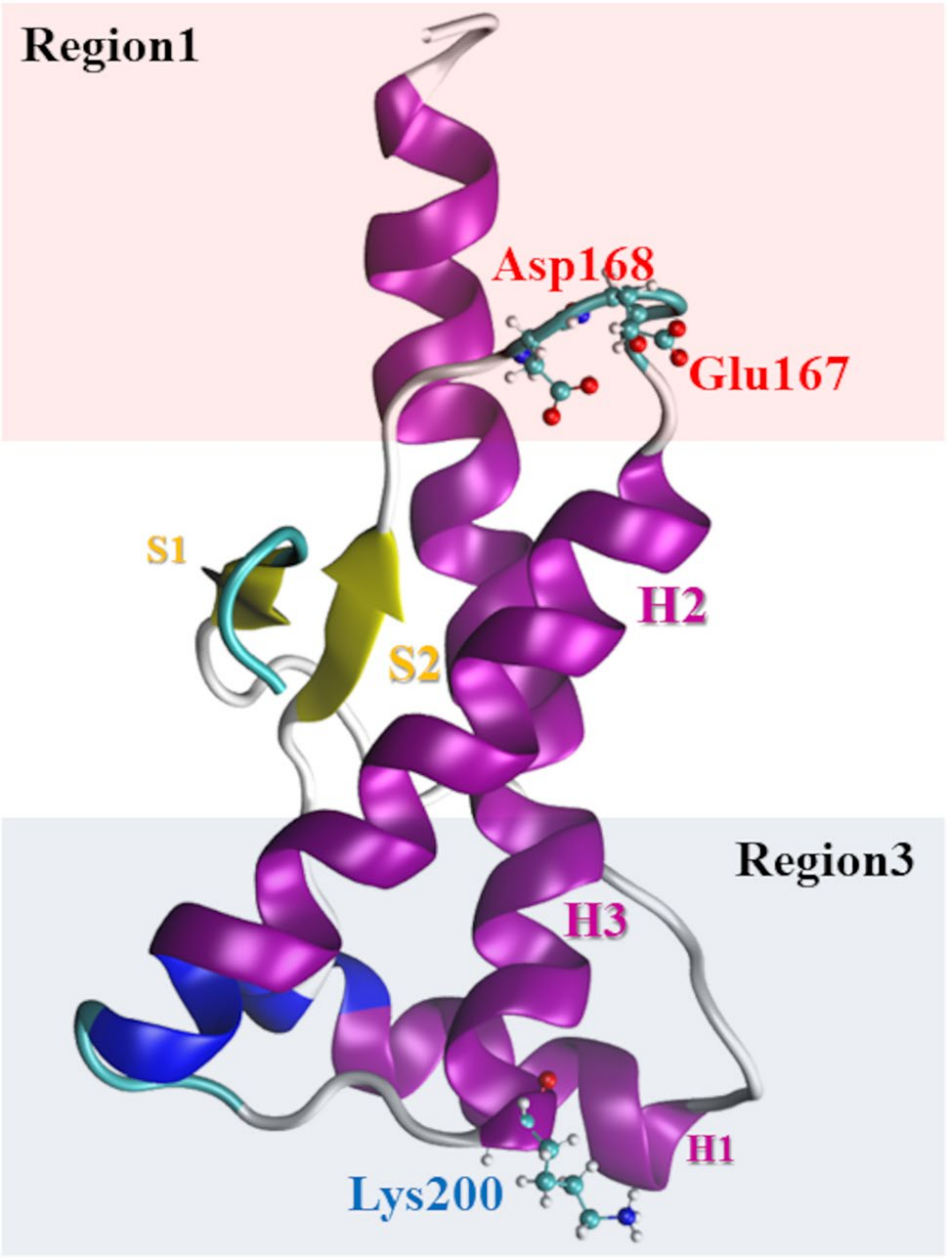
Rendition of the 3D structure of the globular domain (120–231) of PrP-E200K. The electrostatic character of the space surrounding each region is indicated: region1 (pink), negative; region3 (cyan), positive. The mostly contributing charged residues in each region are also shown (ball-and-sticks)

The human prion protein (PrP) [1, 2] is a glycoside-conjugated, membrane-anchored protein characterized by an unstructured N-terminal domain (23–120 segment) and by a folded, often called globular, domain (120–231 segment). This natively folded protein has been identified as the causative agent of severe neurodegenerative diseases due to the capability of degenerating into a neurotoxic and self-replicating amyloid form, which aggregates by producing fibrillar aggregates, i.e., PrP^Sc^ [3]. The recombinant 90–231 segment of PrP has been found to constitute the protease-resistant core of PrP^Sc^ and to gain the same folding of the whole protein, thus, becoming the most employed model of the cellular prion protein [4]. Several NMR studies have shown that point mutations of 90–231 PrP induce negligible modifications to the general folding; nevertheless, such mutations have been also found to impact either the internal stability or the self-aggregation propensity of this protein, thus, providing a rationale to the pathogenicity of these subtle sequence alterations [5, 6]. Interestingly, the N-terminal 106–126 segment has been found to hold per se most of the prionic features which is able to reproduce the PrP^Sc^ effects as induction of apoptosis in neurons, fibrillar formation, resistance to protease K digestion, and induction of the conversion of PrP to PrP^Sc^ [7–10]. Moreover, point mutations occurring on the natively folded domain, i.e., globular domain, seem to be capable of modulating the amyloid propensity of PrP, thus suggesting that some type of structural cross-talk might hold between the two domains. One of the well-known pathogenic point mutations affecting the PrP is represented by the E200K mutation, responsible for a well-characterized familial form of the Creutzfeld-Jakob disease [6, 11]. Recently, we have shown computationally that this mutation may alter the molecular interaction properties of the 120–231 domain of PrP-E200K by enhancing the propensity of this protein to self-assembling [12–14]. Our calculations have unveiled that the E200K mutation alters the charge distribution on the protein surface by inducing an electrostatic complementarity in two regions, named region1 (negative) and region3 (positive) (Fig. 1). The presence of two portions of complementary electrostatic character may act as long-range promoters of the self-aggregation [13, 14]. Based on this outcome, we have postulated that the PrP-E200K protein may undergo early aggregation, where the protein units substantially keep the native, i.e., PrP^C^-like, folding of the 120–231 segment. Molecular dynamics, molecular electrostatic potential (MEP)/molecular interaction field (MIF) analyses, and fragment molecular orbital (FMO) calculations have then provided for corroboration to the early aggregation hypothesis, by evidencing the stability of PrP-E200K dimers obtained through the region1-region3 interaction construct [14].

In this frame, we tentatively propose that the early aggregation of PrP^C^ may be considered a preliminary step to the amyloid conversion: the protein–protein interactions (PPIs) formed in these early aggregates may gradually shape the structure of the assembled protein units by promoting the β-sheet enrichment, and that the hypothesized four-rung or parallel in-register β sheet (PIRIBS) [15] structures of the pathogenic PrP^Sc^ monomers might be not directly yielded by PrP^C^ monomers [16] but, more likely, as a result of a gradual, pro-amyloid maturation of early aggregates.

**Fig. 2:**
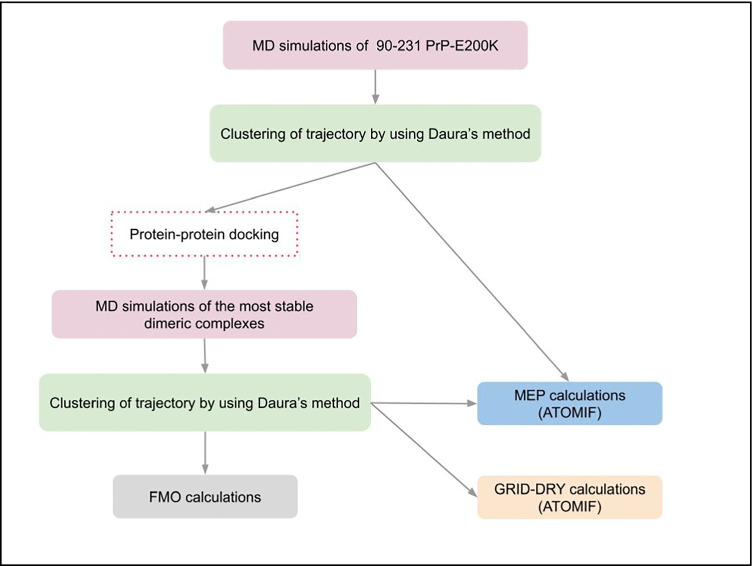
The improved computational workflow applied in this work. We introduced the protein–protein docking step (in red dashed-line box) to identify plausible 90–231 PrP-E200K dimeric complexes

In this paper, with the aim to provide a more reliable model of early aggregation process (homodimerization) of PrP-E200K protein based on complementary electrostatic character of region1 and region3, we consider the extended 90–231 fragments of PrP-E200K. Moreover, to assess whether the fragment 90–120 can affect the MEP of region1 and region3, eventually altering the charge complementary and therefore the protein–protein interaction, we applied a potentiated version of our computational workflow (Fig. 2) previously developed and applied to investigate 125–228 and 120–231 PrP segments [12, 13]. In particular, we included the systematic search of the potential dimeric aggregates by performing protein–protein docking calculations.

MD simulations were performed to sample the 90–231 PrP-E200K conformational space and to provide for a representative subset formed by the most populated structures. The stability of the best scoring protein–protein complexes gained from docking calculations was then assessed by performing MD calculations and an accurate mapping of the PPIs was gained by adopting the FMO/GRID-DRY approach [17].

The effects of the 90–120 fragment on protein MEP and the new models of dimeric aggregates of extended 90–231 PrP-E200K have been discussed and compared with outcomes reported in literature.

The improved computational workflow presented here can be employed to investigate any protein–protein complex where the hydrophobic and electrostatic interactions play a crucial role.

## Methods

### Molecular dynamics and protein–protein docking calculations

The experimental structure of the mutated PrP-E200K [18] was generated from the pdb archive, entry 1FKC. The initial structure of PrP-E200K was instead generated by superposing the 120–231 domain taken from 1FKC onto the corresponding segment of 5YJ5 [19] (90–231 PrP^C^ structure), and by connecting then the 90–119 segment taken from 5YJ5.

The MD simulation of 90–231 PrP-E200K was performed with the Gromacs package [20]. The protein molecule was placed in a cubic box, solvated with TIP3P [21] water molecules, and added by 0.9 g/L of NaCl to gain electrical neutrality as well as to reproduce the physiological ions concentration. The protein systems were simulated in the OPLS-2005 force field [22, 23] starting by a local energy minimization.

Notably, we adopted OPLS/AA to obtain comparable and totally consistent results with our previous studies [12–14, 24] in which we applied the early versions of ATOMIF modules. In particular, the MEP calculated by using the OPLS/AA force field charges has been found to be nearly equivalent to the MEP calculated by using QM charges [12].

After 10 ns of equilibration step at NVT conditions, a production run of 200 ns at NPT conditions was performed. The simulations were carried out in an isothermal/isobaric ensemble, using the velocity rescaling scheme (temperature) and the isotropic Berendsen coupling scheme (pressure) [25] at 300 K. The LINCS constraining algorithm [26] was adopted and the long-range electrostatics was computed by the particle mesh Ewald method [27]. The last 100 ns segment of stable production run trajectory was sampled by extracting one snapshot per 100 ps, to form a set of 1000 protein conformations that were then used for comparative analyses. All further analyses were performed by using suitable Gromacs utilities with the support of either VMD [28] or Maestro [29] graphical interfaces.

Representative subsets of protein conformations were obtained by the employment of the *g_cluster* utility through the method labeled *gromos* [30]. A clustering cut-off of 0.25 nm was employed in the comparison of sampled MD snapshots based on the position of the backbone atoms, and the middle elements of the clusters covering at least 95% of the whole ensemble were used to generate the representative subset of each protein system.

After geometry optimization with Gromacs in the OPLS/AA force field [22, 23], the most significant representative structures of E200K clusters were then used as input for subsequent protein–protein docking calculations by using Rosetta [31, 32] adopting the global docking protocol and considering proteins as rigid bodies. Based on the number of representative conformations obtained per each PrP system (vide infra), three protein–protein docking campaigns were required to evaluate all the possible dimer combinations. To ensure an adequate exploration of the large configurational space of the possible PPIs, we perform 10,000 runs for each docking campaign. The resulting docking poses were ranked by considering the “total score” values, the highest (the most negative) score. In each docking experiment, the structure of a monomer, labeled A, is fixed, while the roto-translational configuration of a second monomer, labeled B, is explored. Based on the Chaudhury benchmarking of the Rosetta scoring [33], we expected to find the most significant E200K dimeric configurations within the five protein–protein complexes with the lowest energy values (“total score” values). The five dimer models with lowest energies, named *d1*, *d2*, *d3*, *d4*, and *d5*, were extracted as the most significant E200K dimeric aggregates.

With the only scope to assess the stability of the PrP-E200K dimers predicted by docking, the *d1–d5* structures were simulated for 100 ns in the NPT ensemble by adopting the same protocols used in the MD simulation of the monomer, including the trajectory clustering to obtain representative subsets of the examined protein–protein complexes. In this case, each dimer subsets was clusterized based on the position of the backbone atoms by applying cut-off values in the range 0.25–0.61 nm. Again, the middle elements of the clusters covering at least 95% of the whole ensemble were minimized in the OPLS/AA force field. After alignment on their principal inertia axes, the optimized E200K dimeric complexes were suitable to ab initio FMO calculations and grid-based analyses.

### Ab initio FMO calculations

The representative structures of each subset obtained by the clustering of MD trajectories of the 90–231 PrP-E200K monomer and dimers were analyzed by means of ab initio fragment molecular orbital (FMO) calculations. In the following, the peptide chains of the two PrP units forming a dimer were labeled with A and B, in accordance with the labeling scheme adopted in protein–protein docking calculations (see “Molecular dynamics and protein–protein docking calculations” section). Each residue is indicated by adding the subscript A/B to the name of the residue (e.g., _A_Lys200, _B_Ser231).

Single-point energy calculations were carried out by using the FMO method [34–37] at the RI-MP2/6-31G* level of theory, implemented in the GAMESS-US program package [38], and by adopting the PCM < 1 > method [39] to describe the solvation effects.

The fragmentation scheme comprised single amino acids, apart from residues involved in disulfide-bond, as Cys179–Cys214, which were considered a single fragment. The fragmentation point was located between Cα and NH group, using the hybrid orbital projection (HOP) treatment for bond detachment [40]. The pair interactions energies decomposition analysis (PIEDA) [41, 42] was performed with the application of bond-detached atom (BDA) corrections [40]. In details, PIEDA permits the decomposition of the pair interaction energy (PIE) in five contributions, i.e., electrostatic (E_es_), exchange repulsion (E_ex_), charge transfer (E_ct+mix_), dispersion (E_disp_), and solvation energies (E_solv_), providing useful information about the nature of the interactions [24].

In order to assess the stability of the E200K dimeric complexes, the FMO binding energy, ∆E^FMO^, was calculated following the procedure reported by Fedorov et al. [37] and briefly summarized in Supporting Information (Note S1).

In the case of E200K dimers, the PIE values estimating the PPIs between chains A and B were collected and named PIE^AB^. The PIE^AB^ values were then recast in either single residue or domain contributions. Secondary structure assignments were performed through the stride [43] analysis of the most representative structure of the E200K monomer (Table S1).

Finally, the stability of alpha-helix secondary structure elements, ∆PIEα, was evaluated via the intra-domain pair interaction energy by computing only the interaction energy within fragments of the same alpha domain, i.e., H1, H2, or H3, as described in Note S1.

### GRID-DRY and MEP analyses

The molecular interaction fields (MIFs) and the molecular electrostatic potential (MEP) of the PrP-E200K models retrieved by MD simulations were calculated adopting the procedure described in the previous works [12–14, 17, 24, 44]. Briefly, by using the GRID method [44], the interaction field is computed as the sum of the interactions of the specific probe with all atoms of the target immersed in the 3D grid. For instance, the uncharged hydrophobic (DRY) probe allows detecting qualitatively/quantitatively the hydrophobic and hydrophilic regions of the target, respectively. Thus, a 3D array of the probe-target interactions can be obtained that corresponds to the MIF.

The GRID-DRY (grid spacing = 0.1 nm) of the representative structures were analyzed to count high-field grid-points, the average and the total MIF per slice (summation of the MIF on all slice points divided or not, respectively, by the number of points).

The computed GRID-DRY MIF was used to study the hydrophobic interaction between the two monomers [17], A and B, of 90–231 PrP-E200K dimeric complexes. All the point of the DRY MIF of the monomer A were enclosed in a sphere equal to each atom’s van der Waals radius, centered on each atom of the second molecule B. Thus, the number of enclosed points within each atom of B, as well as the sum of all interaction energy values, represented a reasonable measure of the hydrophobic interaction between the two monomeric units of the E200K dimer [17]. The hydrophobic energy (HE) associated to protein–protein interaction is then computed.

In a similar way of MIF, MEP is defined as the interaction energy between a positively charged probe (+ 1) located at a given grid point (grid spacing = 0.1 nm) and the atomic charges of the protein, providing information on the presence of positively or negatively charged regions [13, 14].

The MEP of 120–231 and 90–231 PrP-E200K systems was computed by using the classical Coulomb potential in the gas phase (unitary dielectric constant), employed in our previous works [12–14, 17, 24]. Finally, the computed MEP was used to perform pairwise comparisons between 120–231 and 90–231 PrP-E200K protein subsets, using the Carbo index evaluation [24] to assess the impact of 90–120 fragments on E200K MEP.

The GRID-DRY and MEP calculations were performed by using the in-house developed software, ATOMIF [45], and its accurate description will be reported in a further specific article.

## Results

### MD simulations and protein–protein docking: seeking for plausible assembly schemes

The structures of PrP-wt and PrP-E200K retrieved from the protein data bank (PDB, entry 5YJ5 and 1FKC, respectively) were employed to generate the 3D model of the corresponding 90–231 fragment of PrP-E200K mutant. E200K structures were then simulated for 200 ns in an aqueous bulk characterized by an electrolyte concentration of 0.9 g/L (see “Methods” section). The obtained trajectories showed how the system gained equilibration within the time segment 0–100 ns, with stable RMSD profiles in the lasting 100–200 ns segment of trajectories (Fig. S1).

The root mean square fluctuations (RMSFs) analysis indicates that the 90–120 segment and C-term domain are the only protein regions characterized by a significant fluctuation during the simulation (Fig. S2).

The trajectory of E200K was clusterized based on the atomic coordinates of charged residues to obtain representative subsets of protein conformations (Table S2) which is the same criterion applied in the clustering of the 120–231 segment’s simulation [13].

**Fig. 3:**
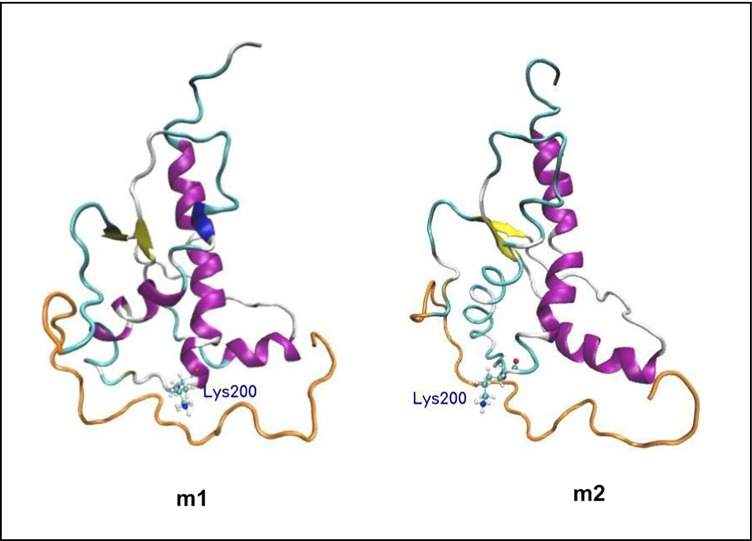
Rendition of the most representative configurations of 90–231 PrP-E200K, *m1* (left) and *m2* (right). The side chain of Lys200 is shown. Fragment 90–120 is also shown (orange)

The representative structures of the top-ranked clusters representing the PrP-E200K were depicted in Fig. 3. In particular, the N-terminus mostly kept an extended conformation by rolling out the manifold formed by H2-H3 loop and H1 helix.

In our previous works [13, 14], we postulated that a possible mechanism of early aggregation of E200K mutant can be ascribed to the interaction between the opposite charged protein regions, named region1 and region3. The positively charged N-terminal (90–120 peptides) is a very flexible domain, characterized by high mobility, with the possibility to affect the electrostatic potential of several protein regions. These considerations led us to assume that the formation of homodimeric complexes cannot be limited to a head-to tail interaction between region1 and region3 and that other regions may be involved in the protein–protein association.

Thus, in order to detect the most reliable homodimeric PrP-E200K complexes, we performed protein–protein docking campaigns involving the most representative conformers extracted from the PrP-E200K trajectory, namely *m1* and *m2* (Fig. 3).

All dimer models obtained from the three possible combinations *m1–m1*, *m1–m2*, and *m2–m2*, were ranked based on the total energy (see “Molecular dynamics and protein–protein docking calculations” section) to finally retain five complexes with the lowest energy. Interestingly, the five models at lower energy, named *d1*, *d2*, *d3*, *d4*, and *d5*, were all members of the *m1–m1* family, (Figs. S3 and S4).

The protein–protein interface of the highest scored complex, *d1*, (Fig. S3) involves the His111-Leu125 fragment of the chains A and B and, in particular, the Ala118-Gly119-Ala120-Val121-Val122 turn. This structural motive heads forward to the hydrophobic pocket located at the end of H2 and at the beginning of H3 helices, also including residues of the H2-H3 loop of each chain. A and B chains in *d1* are oriented in order to approach region3, close to region1, and characterized by opposite charges. Hence, compared to our previous assembly hypothesis [14], the interaction between region1 and region3 is also maintained in the *d1* model although involving a different asset of PPIs. The *d2*, *d3*, and *d4* poses (Figs. S3 and S4) are characterized by a spatial arrangement of A and B chains similarly to that detected in *d1*, although with some differences, while *d5* showed a different assembly motif (Fig. S4). A more accurate description of the best docking poses is also available in Supporting Information (Note S3).

In order to assess the stability of *d1–d5* complexes, MD simulations of these models were performed. All the dimeric complexes remained associated during the entire production run (100 ns), suggesting that the predicted binding poses can be considered reliable (Fig. S5). However, we noted an increase of the RMSD value only for *d4* due to the fluctuation of 90–120 fragment during the simulation.

Each trajectory was investigated by performing cluster analysis and extracting representative subsets for each of the five examined *d1–d5* dimeric configurations. The subset elements are labeled with *a*, *b*, … etc. according to the decreasing weight (Table S3).

**Fig. 4:**
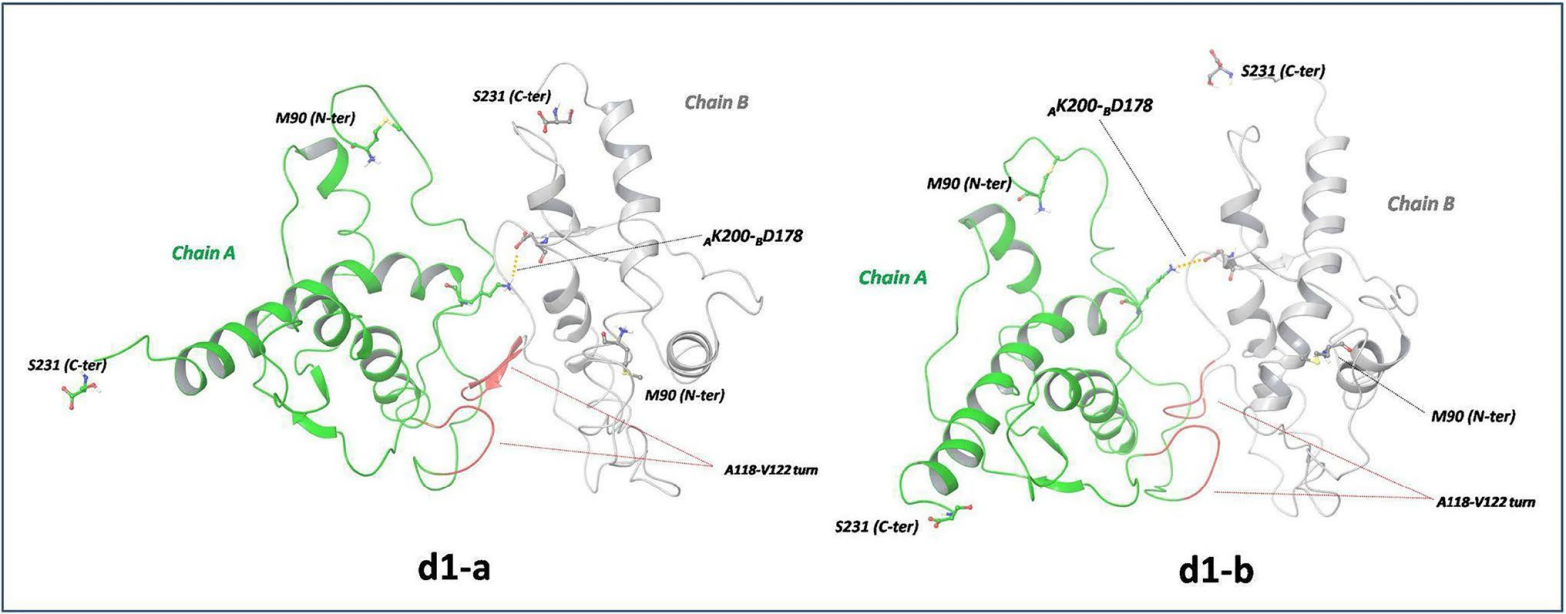
The most representative structures, *d1-a* (weight of 0.92) and *d1-b* (weight of 0.08), of *d1* complex, according to cluster analysis of MD trajectory. The A118-V122 turn motives and H bonds are reported in red and in yellow dashed line, respectively

**Fig. 5:**
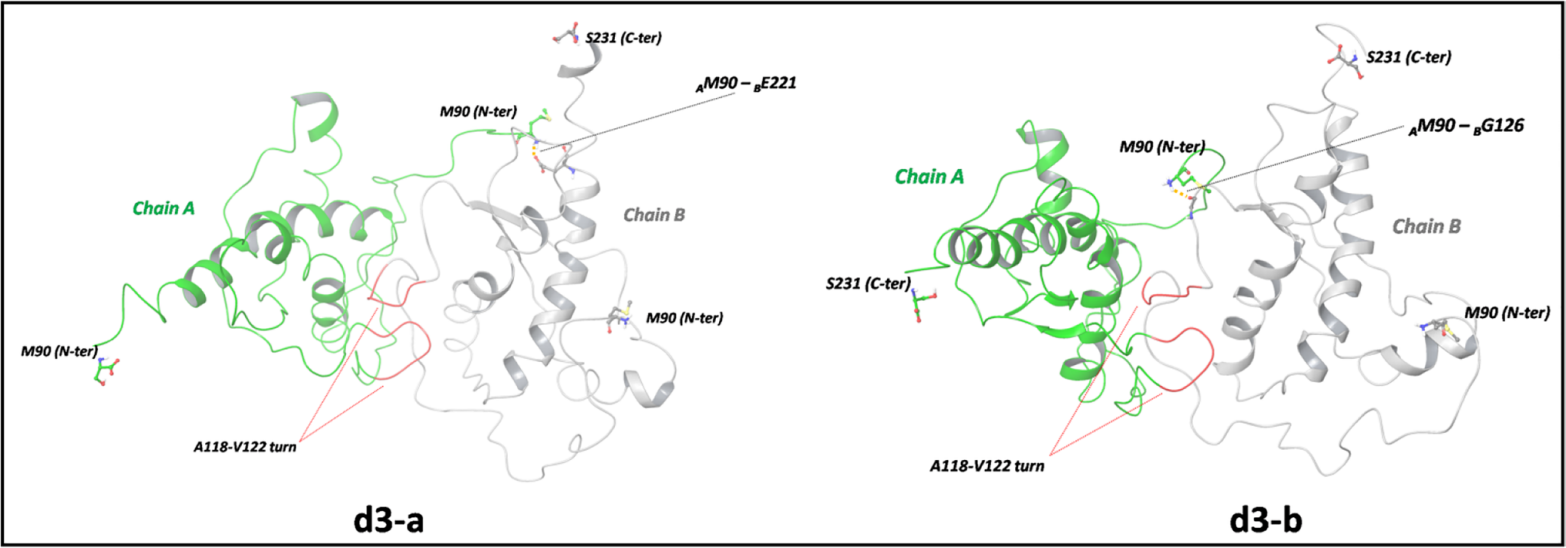
Cartoon models of the most representative structures of the *d3* complexes, *d3-a* (weight of 0.94) and *d3-b* (weight of 0.06), according to cluster analysis of MD trajectory. The A118-V122 turn motives are reported in red

Overall, the MD simulations led to a refinement of the protein–protein interface interactions, with reduction of region1–region3 distance. Indeed, as shown in Fig. 4, the most representative *d1-a* complex (weight of 0.92) is featured by a partial rearrangement of the protein–protein interface in which the turn motif (Ala118-Val122) of chain B still interacts with _A_H2/_A_H2-H3/_A_H3, while the same turn of A heads forward to a different direction with no contact with _B_H2, _B_H2-H3, and _B_H3. Indeed, while the _A_Lys200-_B_Asp178 distance is 3.71 Å, shorter than the corresponding docking value (5.6 Å), _B_Lys200 and _A_Asp178 are extremely distant apart and cannot form any interaction. Conversely, the relaxation process leads to a reduction of the distance between the _A_N-term, placed in the proximity of region3, and _B_C-term located in the region1. The interactions between these two protein segments are detected in the most representative structures of the *d3* complex, *d3-a* and *d3-b*, shown in Fig. 5. In the highest weighted, *d3-a*, the positively charged ammonium group of _A_Met90 forms a strong H bond with _B_Glu221 (Fig. 5). In both A and B chains, the turn His111-Leu125 maintains the interaction motifs previously detected in docking poses.

The most representative structures of *d2*, *d4*, and *d5* maintain essentially the interaction patterns detected in docking calculations (Figs. S6–S8, S9–S11, and S12–S13) with an improvement of a mutual approach of region1 to region3 of two chains. Moreover, in the *d4-c* assembly (Fig. S11), some important contacts are enhanced as the strong ionic interactions involving the interchain residue pairs AGlu221-_B_Met90, _A_Lys101-_B_Ser231, and _A_Gln98-_B_Ser231.

### Fragment molecular orbital insight of the PrP-E200K dimers’ stability

An in-depth examination of the stability of protein–protein interfaces and of the nature of chemical interactions within the structures of PrP-E200K dimers *d1-d5* was performed via ab initio FMO calculations, considering the most representative structures resulting from cluster analysis of MD trajectories (Table S3).

The FMO binding energies, ∆E^FMO^, were estimated with ab initio accuracy to assess the stability of the E200K dimeric complexes (Table 1). Compared to the pair interaction energies (PIE), the ∆E^FMO^ values provide for better estimates of protein–protein binding energies because they include the destabilization polarization and desolvation energies of each free monomer by passing into the dimeric complex.

**Table 1:** Binding energies, ΔE^FMO^ (Note S1, Eq. 2), and pair interaction energies between residues on different chains, PIE^AB^. All values are reported in kcal/mol

Structure	ΔE^FMO^	PIE^AB^
d1	+ 12.9	− 71.4
d2	+ 28.3	− 122.9
d3	+ 17.9	− 212.6
d4	+ 153.3	− 169.2
d5	+ 34.5	− 101.5

All complexes are characterized by positive ∆E^FMO^ values with the lowest ones computed for *d1* and *d3*, with + 12.9 and + 17.9 kcal/mol, respectively, while the highest value of + 153.3 kcal/mol was calculated for *d4*. This large ∆E^FMO^ value suggests that the dimeric complex may not yet have reached a stable conformation of the binding interface reflecting the RMSD profile (Fig. S5). Indeed, during MD simulation, the *d4* complex rearranges the binding conformation to maximize the interactions of the oppositely charged C- and N-terminal domains of the two chains. However, the fluctuation of the flexible 90–120 fragments leads to a constant variation of distances between oppositely charged residues (e.g., _A_S231-_B_M90 and _A_M90-_B_S231), as represented by the *d4-a*, *d4-b*, and *d4-c* structures (Figs. S9–S11). This might significantly affect the magnitude of the FMO pair interaction energy, which is highly sensitive to electrostatic interactions (attractive and repulsive), leading in this case to a less favorable ∆E^FMO^.

Interestingly, according to FMO results, *d1* is the most stable complex as predicted by docking calculations.

It is worth noting that each ∆E^FMO^ value corresponds to the weighted-average of the interchain binding energies computed on each conformer (see Note S1), whose values are reported in Table S4. Hence, for instance, the positive ∆E^FMO^ value computed for *d1* results by combining the positive binding energy of *d1-a* (about + 40 kcal/mol) with the strongly negative value of − 240.3 kcal/mol computed for the lower weighted *d1-b*. The presence of strongly assembled, but low weighted dimers, was ascertained also in other models, such as d1-b, d2-c, d4-c, and d5-b, and indicates that system configurations suitable for initiating the aggregation of PrP-E200K are sampled. As expected, the PIE^AB^ values, providing estimates of the strength of the PPI, were always negative values, thus highlighting the important contribution of the destabilization polarization and desolvation energies to the ∆E^FMO^ values. As reported in Table 1, the strongest interchain interactions were detected in *d3* with − 212.6 kcal/mol, while the corresponding value in *d1* was only − 71.4 kcal/mol. As above stated for the ∆E^FMO^ values, the PIE^AB^ of a single conformer (Table S4) may assume strongly negative values, as in the case of *d4-c* in which we found a huge negative value of − 828.8 kcal/mol, in agreement with the strong H bond interactions, involving oppositely charged chain termini (_A_C-ter-_B_N-ter and _B_C-ter-_A_N-ter), described in the previous section.

The estimated PIE values allowed us to quali-quantify the interaction energy between each residue of A and all residues of B domains, and vice versa. As shown in Tables S5 and S6, the analyses of PIE^AB^ values of interdomain interactions indicate that N-ter, H1, S2-H2, H2, and H3 of both chains A and B are involved in the most attractive protein–protein interactions according to the structural features detected in the docking poses. It is worth noticing that S2-H2 domain includes several critical residues such as Asp167 and Glu168, with a possible role in the prion aggregation process [46]. On the contrary, H1-S2 and C-ter portions are the only domains for which the total PIE^AB^ assumes a positive value (Tables S5 and S6) indicating that for those domains, the repulsive interactions prevail due to the proximity of residues with the same charge.

The analysis of PIE^AB^ values of single residues resulted particularly informative about the contribution of each residue to the stabilization of protein–protein assemblies. Interestingly, we recorded more negative PIE^AB^ values for the residues in the B chain, and, in particular, corresponding to either Asp or Glu. In the case of *d1* (Fig. S14), the most attractive interactions were assigned to Asp178 both in chain A (− 30 kcal/mol) and chain B (− 63 kcal/mol). Other residues contributing to the stabilization of *d1* are Asp144, Glu146, Glu168, Asp202, Glu207, and Glu211, again, displaying more negative per-residue PIE in the chain B residues. These residues are involved in relevant interactions also in *d2*–*d5* complexes (Figs. S15–S18).

The PIE values were also used to assess the stability of α-helix domains H1, H2, and H3, and how it is affected by the assembly. Thus, we calculated the ∆PIE for each α-helix domain of *d1*–*d5* complexes with respect to the corresponding in the E200K monomer, and results are reported for either chain A or chain B in Tables S7 and S8, respectively. The high positive values of ∆PIEs calculated for H2 and H3 indicate that these domains gain instability consequently to the assembly. Interestingly, we also detected negative values of ∆PIEs, specifically for the H1 domains which thus, conversely, is seemingly more stable in the dimer complexes.

### MEP and DRY-MIF analyses

**Fig. 6:**
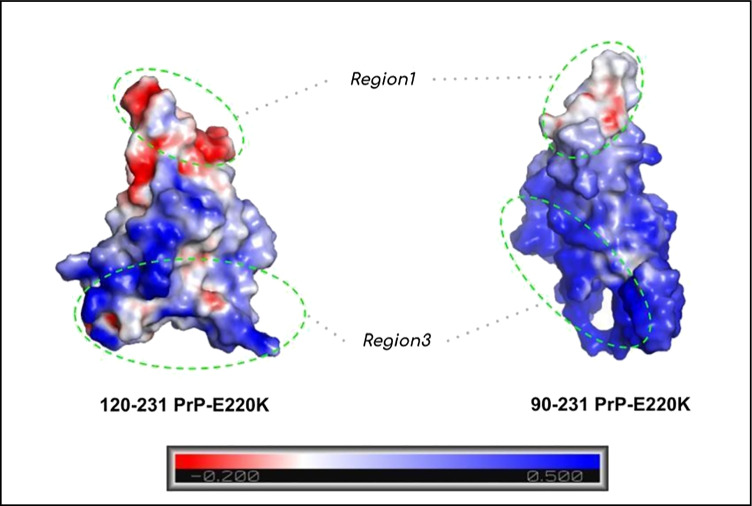
The averaged MEP profiles of 120–231 PrP-E200K (left) and 90–231 PrP-E200K (right). The negatively and positively charged regions are reported in red and blue, respectively. MEPs are computed and reported in arbitrary unit ranging in the − 0.200 up to + 0.500

The representative subsets of 90–231 PrP-E200K formed by two conformations (Table S2 and Fig. 3) were subsequently analyzed in terms of MEP via the mentioned ATOMIF tool [45]. The weighted MEP computed for 90–231 PrP-E200K is displayed in Fig. 6 in comparison with the 120–231 fragment. In both 90–231 and 120–231 fragments, region1 is still characterized by the negative charge region. However, in 90–231 PrP-E200K, the positively charged region, region3, is not limited to 120–231 but assumes a higher extension due to the 90–120 fragment. Therefore, this outcome suggests that the addition of the 90–120 peptides (N-terminal domain) affects the electrostatic potential of the protein surface but region1 and region3 still maintain a negatively and positively charged surface (electrostatic complementarity) which is the main feature of our early aggregation hypothesis.

In order to evaluate in greater details the effect of fragment 90–120 on the MEP of the 90–231 PrP-E200K, the cross-similarity profiles between 120–231 and 90–231 fragments were assessed through the calculation of the Carbò indices along the *x*, *y*, and *z* axis (Fig. S19). As shown in Fig. S20–S22, the lowest electrostatic similarity was identified with the region of the space (slices) containing residues of C-terminal, H1-H2, H3, and N-ter domains. The detected minimum of cross-similarity reflects the different conformations assumed by the 90–120 segment (N-term), characterized by several positively charged residues that can also partially screen the negative charge of C-term domain.

FMO method allows detecting with great precision the electrostatic interactions as salt bridges, H bonds and, in general, polar contacts. These types of interactions are long-ranged and, thus, result to be important especially in the early stage of the protein aggregation by promoting the formation of transient protein–protein “encounter complex” [47, 48]. Thus, the electrostatics interactions play a crucial role in determining the geometry of the initial protein–protein interface and the enthalpic term of free binding energy [49]. A transient complex can be then stabilized mainly by the intervention of hydrophobic interactions because of the great entropic gains they are accompanied [50]. The hydrophobic effect is thus generally reputed the driving force of protein–protein aggregation. Due to the importance of hydrophobic interactions in PPI, we used ATOMIF [44] to detect the most important hydrophobic contacts and identify the protein regions majorly involved in hydrophobic PPIs.

This computational tool was expressly designed to quali-quantify the hydrophobic contacts in either protein–protein interfaces or ligand-receptor complexes [17]. The same approach was applied here to identify the PrP-E200K regions involved in the hydrophobic interactions between units A and B (Table 2), and to compute the hydrophobic energy (HE) related to protein–protein association.

**Table 2:** Hydrophobic energy (HE) between chain A and B within the *d1* − *d5* dimeric complexes of PrP-E200K. All values in kcal/mol

Structure	Hydrophobic interaction energy		
	HE (DRY_B vs A)	HE (DRY_A vs B)	HE
d1	− 11.5	− 15.7	− 27.3
d2	− 4.9	− 13.5	− 18.4
d3	− 11.6	− 13.3	− 24.9
d4	− 8.1	− 11.2	− 19.3
d5	− 21.6	− 18.1	− 39.7

As shown in Table 2, except for *d5*, which is though featured by the strongest interactions, the hydrophobic interactions disclosed by subunit A are stronger, thus suggesting that subunit A may acquire a specific conformation that enhances the hydrophobic contacts. The residues involved in relevant hydrophobic contacts (hydrophobic interaction energies ≤  − 0.9 kcal/mol) are also reported (Table S9). In the *d1* dimer, our analysis unveiled two regions on chain A surface involved in relevant hydrophobic interactions: one is formed by the C-terminus of H2, the N-terminus of H3, and part of the H2-H3 loop, in which some residues strongly interact with hydrophobic field of chain B. The second region is located on the 90–120 fragment where the most important hydrophobic residues are Pro105, Met112, and Ala120. Conversely, the residues of chain B interacting with the hydrophobic field produced by subunit A are located predominantly in the 90–125 fragment and consist of Ala and Leu residues. Interestingly, such an analysis spotlighted those strong hydrophobic contacts that are placed in the amyloidogenic 90–125 segment and in the turn motif His111-Leu125. So, the analysis of hydrophobic contacts between chain A and B suggests that specific interactions between the turn His111-Leu125 of B with the envelope of H2, H2-H3, and H3 domains of A account for most of the hydrophobic interaction energy.

The *d2*–*d4* complexes are also characterized by hydrophobic interactions with these tiled protein regions, although with an opposite location, hence the His111-Leu125 of A interacts with the envelope of H2, H2-H3, and H3 domains of B. On the contrary, in *d5*, the most relevant hydrophobic contacts involve only residues of the His111-Leu125 turn. This evidence reflects the different binding geometry detected for *d5*, compared to the other E200K complexes. It is worth noting that *d5* is the complex with the highest negative HE (− 39.7 kcal/mol, Table 2). Additional descriptions of the hydrophobic contacts are also available in Supporting Information (Note S3).

## Discussion

An atomistic knowledge of the structure and dynamics of the human prion protein (PrP) may help to comprehend the role played by its domains or even single amino acids in the structural alteration process yielding the native PrP^C^ structure to convert in its amyloid, pathogenic *alter ego* PrP^Sc^, and to provide a rationale to the detrimental process triggered by point mutations. A debated point in the PrP amyloid degeneration, caused by either mutations or other stimuli, concerns the articulation of both unfolding and assembly processes undertaken by the globular domain 120–231: does the PrP^C^ protein, as a monomer, unfold before or along the assembly process yielding the PrP^Sc^ structure?

The complete unfolding of a natively folded protein such as the monomeric PrP^C^ is expected to be affected by a large energy barrier because of the unfavorable exposure of its hydrophobic core which can be considered the major contributor to the stability of the 120–231 globular domain. On the other hand, most of the pathogenic point mutants, although encountering an acceleration of PrP^C^ → PrP^Sc^ conversion, are also natively folded and gain the same fold of the wild-type, thus, suggesting that mutants might be affected by a similar energy barrier for the complete unfolding of the monomeric PrP^C^. In our view, an alternative scenario would consider an early aggregation of PrP^C^ in which the intermolecular PPIs may gradually drive the amyloid conversion of the assembled PrP units. We repute that some partial corroboration to such a hypothesis can be found in the literature. Indeed, the formation of PrP oligomers characterized by a still unclear conformational asset of the protein units has been recently proposed as the mesomeric aggregate carrying the strain-specific information [51]. Therefore, the recently postulated existence of a droplet state for many proteins, characterized by high intra and intermolecular variabilities, as well as high local concentrations of the involved protein, seemingly paves the way to the aggregation of PrP^C^ before unfolding [52, 53]. Indeed, the droplet state is reversibly connected to either the monomer (native [53]) or the solid condensed state, the latter being identified with the amyloid state [52]. In this frame, the role of pathogenic point mutations would not be directly related to the capability of destabilizing the native folding of the protein, but rather be connected to the aggregation propensity of PrP^C^.

Interestingly, the self-seeding amyloid aggregation of a truncated version of PrP, i.e., Y145Stop, has been detected to occur via liquid–liquid phase separation, thus, corroborating the hypothesized concomitance of aggregation and amyloid conversion [54].

**Fig. 7:**
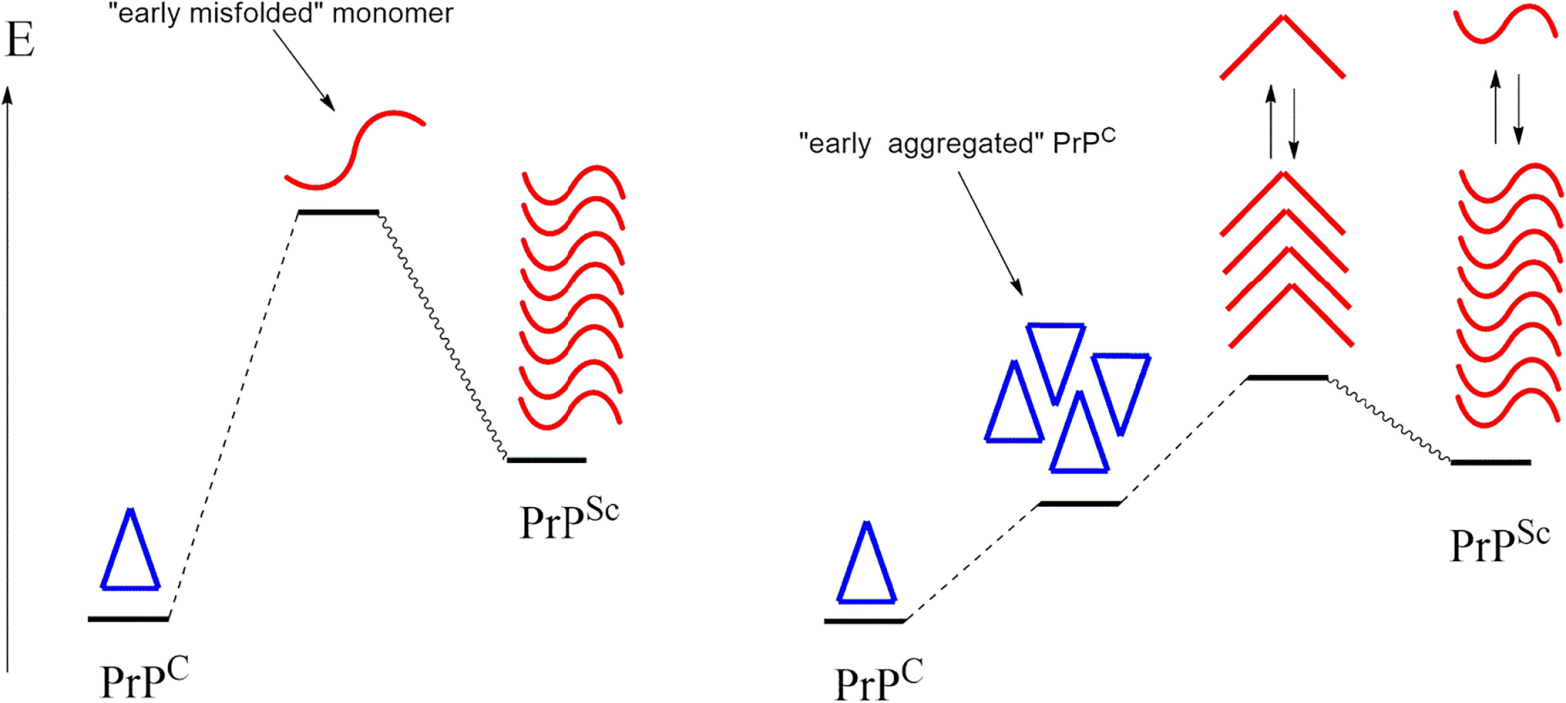
Two alternative scenarios of the PrP^C^ → PrP^Sc^ conversion. In the early unfolding scenario (left), PrP^C^ (triangle) undergoes complete unfolding (sigmoid line) before amyloid aggregation. In the early aggregation scenario (right), PrP^C^ forms aggregates (triangles) that evolve by the gradual unfolding of the protein units (sigmoid lines) before completion of the amyloid aggregation. Notice that either partially or completely misfolded monomers of PrP may coexist in equilibrium with their aggregates

Here, we assumed the early aggregation scenario (Fig. 7) to investigate how the extended pathogenic mutation 90–231 PrP-E200K may alter the molecular interaction properties of PrP. At this purpose, we employed an improved computational methodology developed by us [12] and based on the combination between molecular dynamics (MD), grid-based, and fragment molecular orbital (FMO) approaches, to provide for a quali-quantitative assessment of the molecular properties that control the self-aggregation propensity of protein systems [13, 14]. The ATOMIF tool [45], working together with the GRID software [44], has been expressly designed to implement the proposed multi-layered computational methodology, and suitable to a parallel calculation environment that permits this tool to be employed in the investigation of even large molecular systems.

We extended our computational methodology to the 90–231 segment of PrP-E200K that includes the unstructured 90–120 portion attached to the 120–231 globular domain. Thus, we investigated how the 90–120 fragment can affect the protein MEP and therefore the homodimeric association on the base of the charge complementary region1–region3 previously reported for 120–231 fragment. For this purpose, we improved our methodology by performing extensive docking calculations to retrieve plausible protein–protein assemblies and highlight the PrP-E200K segments majorly involved in the self-aggregation.

As reported elsewhere by us [13], region3 of the 120–231 segment of PrP-E200K is characterized by positive electrostatic character so that the approach of the 90–120 segment, bearing several positively charged residues, reinforces its positive electrostatic character. Hence, 120–231 and 90–231 fragments of PrP-E200K presents an analogous electrostatic complementarity, with a positively charged region (now including the 90–120 segment) and a negatively charged C-terminal region. These two regions of the 90–231 PrP models closely resemble the region1 and region3 of the 120–231 PrP reported by us [13], with the former region being basically the same, while the region3 of 90–231 PrP is differently shaped by the conformation of the 90–120 segment.

Protein–protein docking calculations were then performed to identify and rank plausible self-assembly models of PrP-E200K. The five top-ranked models of dimers obtained by docking were subsequently simulated to better assess the effects of dynamics and water bulk on to their stability. The protein–protein assemblies predicted by docking were substantially confirmed by MD calculations with only slight rearrangements detected at the interunit interface; hence, we limit the discussion to only the *d1*–*d5* models gained by the MD simulations.

The analysis of *d1*–*d5* structures indicates that the formation of dimeric complexes of PrP-E200K can effectively occur via region1–region3 interface suggesting that the complementary electrostatic character of region1 and region3 may play a crucial role in self-assembly of PrP-E200K as previously reported for 120–231 PrP-E200K fragments [13, 14]. It is known that electrostatic interactions play a special role in kinetic of protein–protein association acting as long-range forces. They favor and stabilize the formation of the encounter complex and the following transition state, promoting a fast protein–protein association [49, 55]. Indeed, it has been proposed that the transition state for protein–protein association is stabilized by electrostatic interactions. Although the structure of the encounter complex resembles the structural motifs of the final complex, its protein–protein interface is still solvated or partially solvated. Therefore, the formation of specific short-range interactions is affected by an energy barrier due to the concomitant structural rearrangements and desolvation [55]. Accurate studies on the binding energy of protein associations indicated that the electrostatic contributions are thermodynamically unfavorable at short distances, thus leading to a positive ∆G value for the protein–protein binding [49, 56]. Therefore, to make the protein–protein association a thermodynamically feasible process, the inclusion of hydrophobic interactions is crucial. Notably, the calculated ∆E^FMO^ values for the 90–231 PrP-E200K dimerization indicate that the dimer is higher in energy compared to the separated PrP units, in line with the above-mentioned evidence. All dimeric complexes are characterized by positive aggregation energies with the most favorable (less positive) ∆E^FMO^ values computed for *d1* and *d3* of only + 12.9 and + 17.9 kcal/mol, respectively. To precisely interpret these outcomes, it must be recalled that FMO poorly describes the hydrophobic contacts and entropic contributions, especially when they are associated with the desolvation process. Therefore, the HE of PrP-E200K dimers computed via the ATOMIF tool indicated that the hydrophobic contacts are always favorable (Table 2). Thus, by following the criterion adopted by other authors [56], we estimated the total binding energy, E^tot^, by the summation of ∆E^FMO^ and the HE and assessed favorable (negative) binding energies for 4 out of 5 ligands (Table 3).

**Table 3:** Total binding energy, E^tot^, obtained as summation of the ΔE^FMO^ and HE weighted values computed by FMO and GRID-DRY methods, respectively, for dimeric complexes *d1* − *d5*. All energy values are reported in kcal/mol

Structure	ΔE^FMO^	HE	E^tot §^
d1	+ 12.9	− 27.3	− 14.4
d2	+ 28.3	− 18.4	− 9.9
d3	+ 17.9	− 24.9	− 7.0
d4	+ 153.3	− 19.3	+ 134.0
d5	+ 34.5	− 39.7	− 5.2

^§^ E^tot^ = ΔE^FMO^ + HE

Interestingly, except for *d4*, the analysis of the E^tot^ indicates that the stability of dimeric complexes follows the order *d1* > *d2* > *d3* > *d5* in agreement with relative energy ranking obtained by docking calculations.

This outcome also suggests that the combination of FMO method with GRID-DRY procedure may represent a promising approach to evaluate the electrostatic and hydrophobic interactions in PPI, and therefore to address the stability of protein–protein association where these forces play a crucial role.

The analysis of the protein–protein interfaces in the *d1*–*d5* models reveals the importance of both electrostatic and hydrophobic forces. Indeed, the interfacing between two PrP-E200K units occurs mainly via short-ranged hydrophobic contacts (Table S10) and only a few electrostatic contacts were unveiled, with the major involvement of residue Lys110, Asp178, and Glu221 (Table S10). Interestingly, the importance of Asp178 in the self-aggregation of PrP had been already proposed by others [57], while, more recently, we also identified Asp178 within a potential druggable site of the 120–231 segment of PrP-E200K hit by charged-based inhibitor [24]. Therefore, the S2-H2 loop region had been also indicated to be targeted by cationic molecules such as tetrapyrrole [Fe(III)-TMPyp] that may act as pharmacological chaperones, stabilizing the cellular fold of PrP [58, 59].

DRY MIF results confirm that the interface residues mostly involved in the hydrophobic PPIs resulted to be alanine, glycine, threonine, methionine, and lysine, particularly placed between the turn His111-Leu125 of one unit and the envelope of H2, H2-H3 loop, and H3 domains of the other unit. Although these types of residues are not frequently detected in protein–protein interface hot-spots, alike tryptophan (21%), arginine (13.3%), and of tyrosine (12.3%) [60], a study performed on protease/inhibitor complexes by Krystek et al. [61] indicated that alanine, proline, glycine, and cysteine accounted for a large percentage of residues at the contact surface, supporting the reliability of our results.

The major involvement of the 90–120 domain in the PrP-E200K assembly is confirmed by the high interfacing frequencies detected for the residues within the 110–125 segment. The protein portions centered on Pro165 and Lys185 also present appreciable interfacing frequencies, thus, confirming the importance of S2-H2 and H2 domains in the PrP-E200K assembly.

The analysis of PIE^INT^ values for *d1*–*d5* models computed by FMO method unveiled an unexpected pattern of interunit interactions: attractive interactions (negative PIE^INT^s) are ascribed to negatively charged residues, whereas repulsive interactions (positive PIE^INT^s) are ascribed to positively charged residues. This outcome can be related to a great number of positively charged residues in 90–120 fragment leading to an extended positively charged region and therefore to a local repulsion when two 90–231 PrP-E200K units interact.

The FMO analyses were also performed to assess the stability of secondary structures in response to the PrP-E200K dimerization. In this case, calculations evidenced that while H1 is somewhat stabilized, both H2 and H3 helices are clearly destabilized upon dimerization. This result is in full agreement with the amyloidogenesis scenario featured by the early aggregation of the PrP protein, in which the self-aggregation of PrP triggers the detriment of its native folding.

Finally, our data corroborate the possible critical role of 90–120 fragment in the early aggregation of PrP-E200K, due to its involvement not only in important ionic interactions, as indicated by FMO results, but also hydrophobic interactions as highlighted by GRID-DRY analyses. On the contrary, the hydrophobic spot proximal to the tyrosine triad Tyr169, Tyr225, and Tyr226 in the PrP-E200K monomer was found to be not involved in any hydrophobic interaction at the protein–protein interface of PrP-E200K dimer, although their possible intervention in the stabilization of higher aggregates cannot be totally ruled out.

The multilayered procedure applied in this work can be improved for investigating unfolded protein by adopting specific FFs as CHARMM36m [62], a99SB-disp [63], and GROMOS 53a6 [64] that demonstrated to be suitable for conformational sampling of intrinsically disordered proteins as evidenced by recent works. For instance, the a99SB-disp FF, developed to capture the dynamics of both folded and disordered proteins, has been applied to study the V136R154Q171 mutant of the full-length PrP (residues 22–234) to predict the possible interdomain interactions involved in the misfolding process [65]. GROMOS 53a6 FF has been recently used (i) to address the early misfolding events of V210I-PrP mutant identifying possible aggregation-prone regions [66] and (ii) to study the aggregation process of 18 b-rich H2H3 fragments of the ovine PrP (H2H3-OvPrP^Sc^) by performing atomistic molecular dynamics simulations in the sub-microsecond time-range [67].

Based on this evidence, part of our future research activities will be devoted to applying those specific FFs and eventually enhanced sampling methods to further improve the accuracy of our computational protocol.

## Conclusions

To resume, we presented a detailed computational analysis of the dynamics and molecular interaction properties of the 90–231 segment of PrP-E200K, a pathogenic mutant of the human prion protein. The combination of classical molecular dynamics simulation with grid-based analysis, previously employed to investigate the aggregation propensity of 120–231 segments of PrP-E200K, was here extended and consolidated by including protein–protein docking calculations. Our results indicate that the early aggregation scenario, based on electrostatic complementarity between region1 (negative) and an extended region3 (positive) of two monomer units, is plausible and may explain the higher aggregation propensity of the E200K.

In particular, the self-aggregation of PrP-E200K is initiated by the electrostatics, determining either long-ranged interactions between two interacting units or sculpting the approaching protein domains. Interestingly, positively and negatively charged residues seem to play different roles in the control of the PrP-E200K aggregation: the former mainly being repulsive, probably because of the high number of positive residues placed at the protein–protein interface. On the other hand, the hydrophobic effect can be considered the most important driving force in the formation of PrP-E200K homodimeric complex, although in the early aggregation, we majorly identify the involvement of alanine, glycine, threonine, methionine, and lysine. These residues operate only an initial stabilization of the early aggregates, while being gradually encompassed by other hydrophobic residues during a sort of maturation in which the aggregated PrP-E200K units slowly transit to their unfolded structure. The destabilization of the H2 and H3 helices domains in response to the assembly is a further corroboration to this early aggregation scenario. The detailed picture of the PrP-E200K assembly herein presented might be supportive in either the design of site-directed mutagenesis experiments or the interpretation of the pathogenic role of other mutations of the prion protein.

## Supplementary Information

Below is the link to the electronic supplementary material.

Supplementary file1 (PDF 2923 KB)

## Data Availability

The 3D coordinates of *m1*, *m2*, and of all the *d1*–*d5* structures reported in this study are also available in the pdb file format at the following link https://doi.org/10.5281/zenodo.6584362.

## Code availability

ATOMIF is an open-source software developed in Python by Loriano Storchi used to computed Carbo index profiles and to quali-quantify the hydrophobic and hydrophilic regions of a molecule (https://github.com/lstorchi/atomif).
