# Supplementary material for: Homodimeric complexes of the 90–231 human prion: a multilayered computational study based on FMO/GRID-DRY approach
Source: J Mol Model. 2022 Aug 2;28(8):241. doi: 10.1007/s00894-022-05244-2 (PMC9345805; doi:10.1007/s00894-022-05244-2)
Supplement: Supplementary file 1 — Supplementary file1 (PDF 2923 KB) [file 894_2022_5244_MOESM1_ESM.pdf]

Supporting Information

Homodimeric complexes of the 90-231 human Prion: a Multilayered Computational Study based on FMO/GRID-DRY approach.

Roberto Paciotti[1,*], Loriano Storchi[1,2], and Alessandro Marrone[1]

[1]University "G d'Annunzio" of Chieti-Pescara, Department of Pharmacy, Chieti, Italy.

[2]Molecular Discovery Limited, Middlesex, London, United Kingdom.

* Corresponding author: r.paciotti@unich.it

Contents

Note S1 *Ab initio FMO calculations - details* 4

Table S1 Secondary structures partition of PrP-wt and PrP-E200K used in this work 5

Fig. S1 The root mean square deviation (RMSD) of the backbone coordinates of the 90-120 (red) and 121-231 (green) fragments of PrP-E200K. The RMSF profile of the whole protein (90-231) is reported in black. 6

Table S2 Results of clustering analyses performed on the 100-200 ns trajectories of 90-231 PrP-E200K...... 7

Note S2 *Protein-protein docking: PrP-E200K binding poses* 8

Fig. S3 The lowest energy of dimeric E200K complexes (d1 and d2) obtained by performing protein-protein docking. The relative total energy scores are reported in parenthesis. The A118-V122 turn motives are red colored and H bonds are reported by using a yellow dashed line........ 9

Fig. S4 The lowest energy of dimeric E200K complexes (d3, d4 and d5) obtained by performing protein-protein docking. The relative total energy scores are reported in parenthesis. The A118-V122 turn motives are red colored 10

Fig. S5 The root mean squared deviation of the backbone coordinates of 90-231 PrP-E200K dimers, namely d1, d2, d3, d4 and d5, reported in red, black, green, blue and violet line, respectively.................................. 11

Table S3 Results of clustering analysis of E200K dimeric complexes.. 11

Fig. S6 Structure of d2-a E200K dimeric complex, the most representative structure of MD trajectory of d2 according to cluster analysis. The A118-V122 turn motives are red colored... 12

Fig. S7 Structure of d2-b E200K dimeric complex, resulting from cluster analysis of MD trajectory. The A118-V122 turn motives are red colored 13

Fig. S8 Structure of d2-c E200K dimeric complex, resulting from cluster analysis of MD trajectory. The A118-V122 turn motives are red 14

Fig. S9 Structure of d4-a E200K dimeric complex, the most representative structure of MD trajectory of d4 according to cluster analysis. The A118-V122 turn motives are red colored... 15

Fig. S10 Structure of d4-b E200K dimeric complex, resulting from cluster analysis of MD trajectory. The A118-V122 turn motives are red colored 16

Fig. S11 Structure of d4-c E200K dimeric complex, resulting from cluster analysis of MD trajectory. The A118-V122 turn motives are red colored and H bonds are reported by using yellow dashed line 17

Fig. S12 Structure of d5-a E200K dimeric complex, the most representative structure of MD trajectory of d5 according to cluster analysis. The A118-V122 turn motives are red colored and H bonds are reported by using a yellow dashed line 18

Fig. S13 Structure of d5-b E200K dimeric complex, resulting from cluster analysis of MD trajectory. The A118-V122 turn motives are red colored 19

Table S4 Binding energies, ΔE^{FMO}, and pair interaction energies between residues of the two chains A and B, PIE^{AB}, of the most representative structures resulting from the clustering analysis of E200K complexes 20

Fig. S14 Weighted PIE values computed for d1, between residues of chain A and the whole chain B (blue bar) and vice versa (red bar). The most attractive interactions were assigned to Asp178 both in chain A (-30 kcal/mol) and chain B (-63 kcal/mol): the $_{A}$Asp178 establishes an attractive interaction with $_{B}$Arg208 (-8 kcal/mol) while $_{B}$Asp178 strongly interacts with $_{A}$Lys200 (-19 kcal/mol), in agreement with the geometrical features discussed above. Another important residue is $_{B}$Asp167, which participates in the attractive interaction with $_{A}$Lys104 (-10 kcal/mol), and other residues contributing to the stabilization of d1 are Asp144, Glu146, Glu168, Asp202, Glu207 and Glu211, again, displaying more negative per-residue PIE in the chain B residues 21

Fig. S15 Weighted PIE values computed for d3, between residues of chain A and the whole chain B (blue bar) and vice versa (red bar) 22

Fig. S16 Weighted PIE values computed for d2, between residues of chain A and the whole chain B (blue bar) and vice versa (red bar) 23

Fig. S17 Weighted PIE values computed for d4, between residues of chain A and the whole chain B (blue bar) and vice versa (red bar) 24

Fig. S18 Weighted PIE values computed for d5, between residues of chain A and the whole chain B (blue bar) and vice versa (red bar) 25

Table S5 weighted PIE values (in kcal/mol) between secondary structure domains of chain A with the whole protein B. PIEs are referred to d1-d5 complexes.. 26

Table S6 weighted PIE values (in kcal/mol) between secondary structure domains of chain B with the whole protein A. PIEs are referred to d1-d5 complexes 26

Table S7 Weighted relative intra-domain interaction energies,ΔPIE, of H1, H2 and H3 computed between E200K and *chains A* of E200K-dimers. In details, H3 domain presented the highest ΔPIE values of +183.7 (chain A of d5), +179.6 (chain A d2), +162.4 (chain A of d1), +151.1 (chain B of d5) and +125.4 (chain B of d1) kcal/mol 27

Table S8 Weighted relative intra-domain interaction energies, ΔPIE, of H1, H2 and H3 computed between E200K and *chains B* of E200K-dimers 27

Fig. S19 position of xyz origin with respect to 90-231 PrP-E200K structure (coordinates) used for MEP and DRY MIF calculations (ATOMIF) 28

Fig. S20 Weighted Carbo MEP cross-similarity profile of the 90-231 and 120-231 PrP-E200K segments, computed along the x axis (see Fig. S19 as reference). The weighted standard deviation is also reported ... 29

Fig. S21 Weighted Carbo MEP cross-similarity profile of the 90-231 and 120-231 PrP-E200K segments, computed along the y axis (see Fig. S19 as reference). The weighted standard deviation is also reported ... 30

Fig. S22 Weighted Carbo MEP cross-similarity profile of the 90-231 and 120-231 PrP-E200K segments, computed along the z axis (see Fig. S19 as reference). The weighted standard deviation is also reported.... 31

Note S3 *DRY MIF and hydrophobic contacts analysis – additional details* 32

Table S9 Hydrophobic interaction energies, in kcal/mol, of residues involved in the most significant hydrophobic contacts (hydrophobic energies ≤ -0.9 kcal/mol) 32

Table S10 PrP-E200K residues at the d1-d5 interfaces. Residues in either A or B unit placed within less than 3.0 Å from the interfacing B or A unit, respectively, are reported in columns A|B and A|B. Ionizable residues or groups, i.e., Asp, Glu, Arg, Lys, His, C-ter and N-ter, in either A or B unit placed within less than 6.0 Å from the interfacing B or A unit, respectively, involved in long-ranged electrostatic contacts, are reported in columns ∓A|B 33

Note S4 *The mdp file used for NPT simulation with Gromacs* 34

Note S1 *Ab initio FMO calculations - details*

In order to assess the stability of the E200K dimeric complexes the FMO binding energy, ΔE^{FMO}, was calculated following the procedure reported by Fedorov et al [37]. In details, the ΔE^{FMO} value of a E200K dimeric complex (*D*) resulting from a combination of two E200K monomers (M_A and M_B) was calculated according the following equations:

$$M_A + M_B \rightarrow D \quad (1)$$

$$\Delta E^{FMO} = E_D^{FMO} - \left(E_{M_A}^{FMO} + E_{M_B}^{FMO}\right) \qquad (2)$$

Each E^{FMO} values is computed as described by eq. 3:

$$E_M^{FMO} = \sum E' + \sum PIE^{IJ} \qquad (3)$$

$$E' = E_i + E_{sol} \qquad (4)$$

$$PIE^{IJ} = E'_{IJ} - E'_I - E'_J + Tr(\Delta D^{IJ} \Delta V^{IJ}) \qquad (5)$$

where E' is the internal energy of each monomer that can be divided into the internal energy of solute E_i and PCM solvation energy E_{sol} (eq. 4). The PIE^{IJ} within the FMO2 method is defined by eq. 5 where ΔD is the density transfer matrix for dimer IJ and V is the matrix of the contribution of a third fragment to the electrostatic potential acting upon dimer IJ [37].

The PIE^{IJ} value can be divided into five energy components by PIEDA, as shown by the following equation:

$$PIE^{IJ} = E_{es} + E_{ex} + E_{ct} + E_{disp} + E_{solv} \quad \text{(eq. 6)}$$

where the terms E_{es} ,E_{ex}, E_{ct} , E_{disp} and E_{solv} refer respectively to electrostatic, exchange repulsion, charge transfer, dispersion and solvation energies [42], providing useful information about the nature of the interactions.

In the case of E200K dimers the PIE values estimating the protein-protein interactions between chains A and B, were collected and named PIE^{AB}. The PIE^{AB} values were then recast in either single residue or domain contributions. Secondary structure assignments were performed by the Stride analysis [43] of the most representative structure of the E200K monomer as reported in Table S1.

Moreover, the stability of alpha-helix secondary structure elements was evaluated via the intra-domain pair interaction energy by computing only the interaction energy within fragments of the

same alpha domain, i.e., H1, H2 or H3. The intra-domain PIE values computed for E200K dimers were then compared with the ones for the E200K monomer by defining ΔPIEα:

$$\Delta PIE\alpha = {}_{A}PIE\alpha - {}_{E200K}PIE\alpha \quad (7)$$

positive or negative values of ΔPIEα indicate decreasing or increasing domain stability, respectively.

ΔE^{FMO}, PIE^{AB} and ΔPIEα values were finally refined computing the corresponding w_i-weighted values according to eq. 8:

$$\Delta E_{D}^{FMO} = (\Delta E_{D1}^{FMO} x\, w_1) + (\Delta E_{D2}^{FMO} x\, w_2) + \cdots + (\Delta E_{Di}^{FMO} x\, w_i) \quad (8)$$

where w_i is the weight obtained through the clustering of MD trajectory of the systems.

Table S1 Secondary structures partition of PrP-wt and PrP-E200K used in this work

Protein domain	Protein residues	Structural features
N-ter	90-128	Unstructured and flexible domain
S1	129-130	β-sheet
S1-H1	131-143	Loop, flexible domain
H1	144-145	α-helix
H1-S2	153-161	Loop, flexible domain
S2	162-163	β-sheet
S2-H2	164-174	Loop
H2	175-189	α-helix
H2-H3	190-200	Loop
H3	201-224	α-helix
C-ter	225-231	Flexible domain

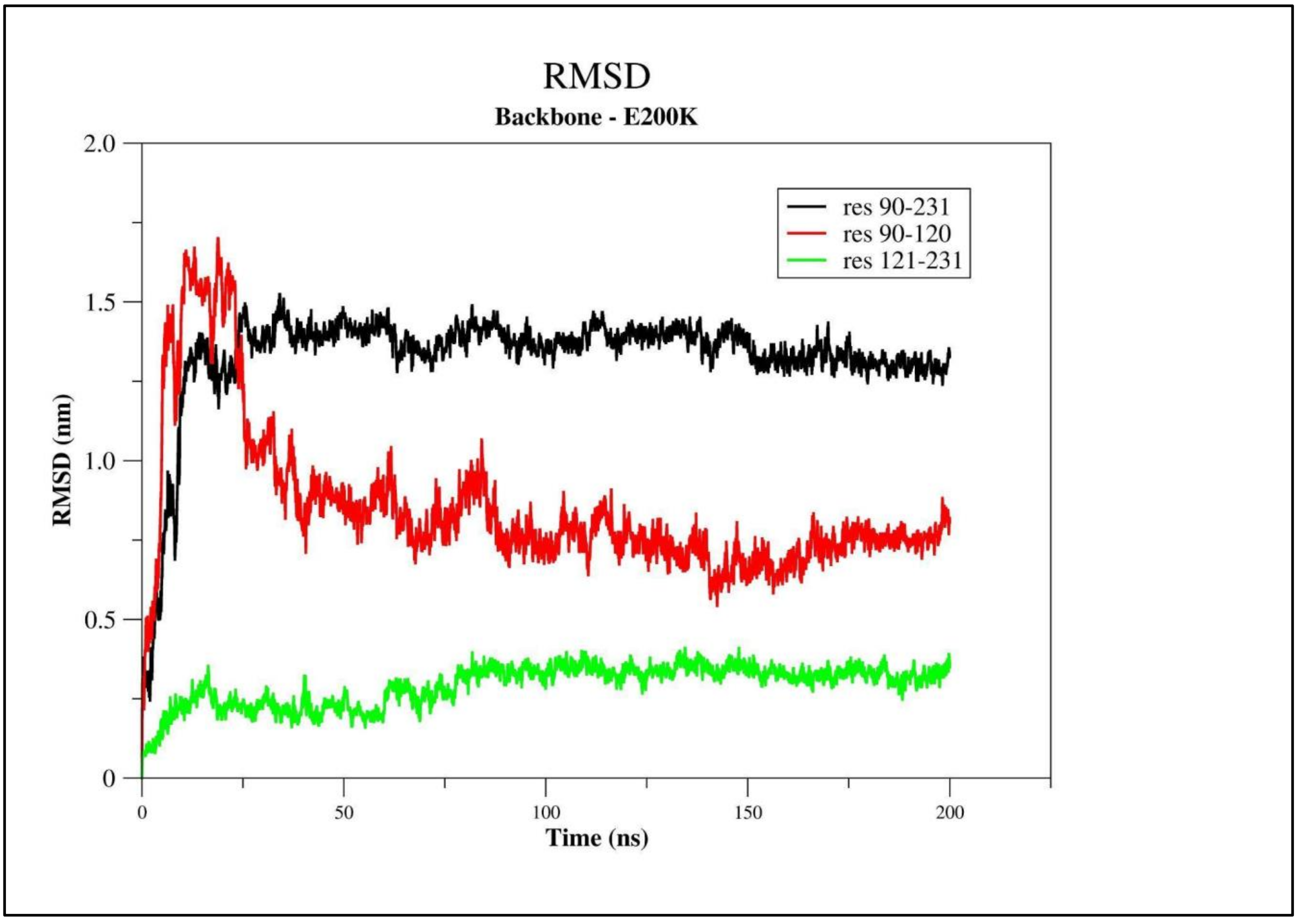

Fig. S1 The root mean square deviation (RMSD) of the backbone coordinates of the 90-120 (red) and 121-231 (green) fragments of PrP-E200K. The RMSF profile of the whole protein (90-231) is reported in black

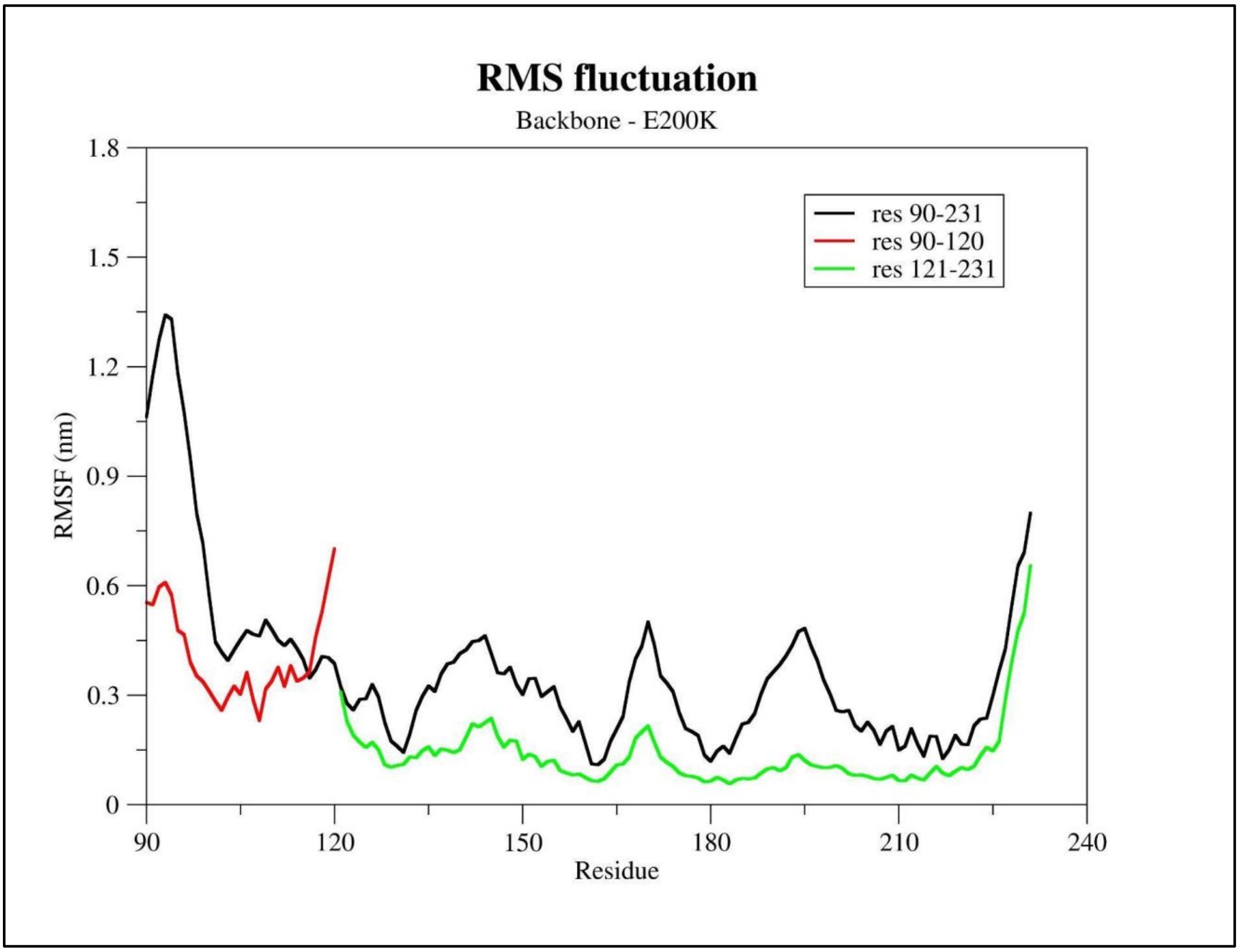

Fig. S2 The root mean square fluctuations (RMSF) of the backbone coordinates of the 90-120 (red) and 121-231 (green) fragments of PrP-E200K. The RMSF profile of the whole protein (90-231) is reported in black

Table S2 Results of clustering analyses performed on the 100-200 ns trajectories of 90-231 PrP-E200K

protein	#cluster	weights
90-231 PrP-E200K	2	0.86, 0.14

Note S2 *Protein-protein docking: PrP-E200K binding poses*

The highest scored complex, **d1**, (Fig. S3) is characterized by the chains A and B interacting with high symmetry. The protein–protein interface involves the His111-Leu125 fragment and, in particular, the Ala118-Gly119-Ala120-Val121-Val122 turn of each chain heads forward to the hydrophobic pocket located at the end of H2 and at the beginning of H3 helices, also including residues of the H2-H3 loop. Thus, the A and B chains in **d1** are oriented in order to approach the region3 close to region1 characterized by opposite charges. Hence, compared to our previous assembly hypothesis [13, 14], the interaction between region1 and region3 is also maintained in the **d1** model although involving a different asset of protein-protein interactions. The high symmetry of the complex is also highlighted by the distance of $_{A}$Asp178-$_{B}$Lys200 and $_{B}$Asp178-$_{A}$Lys200 that is 5.6 Å for both the interactions.

The **d2** pose (Fig. S3) is characterized by a disposition of A and B chains similar to that detected in **d1**, although with some evident differences. The chain A interacts basically via the turn motif Ala118-Val122 involved in the protein-protein interface (PPI), whereas chain B interacts by means of S1-H1 (Ala133), S1 (Leu130) and H2 (Gln186).

The poses **d3** and **d4** (Fig. S4) showed a PPI similar to that found in **d1** but with a lesser involvement of H3 residues, and with the His111-Leu125 fragment of one chain interacting with residues H2 (Ile182-Thr192) of the other one.

At variance from the symmetrical **d1-d4** models, dimer **d**5 is instead not symmetrical with the region1 of chain A close to the region3 of chain B, but with the negatively charged region1 of B pointing away from region3 of A (Fig. S4).

Interestingly, the 90-120 fragment is always involved in PPIs in any **d1-d5** dimers, in particular by means of His111- Leu125 residues. In detail, an important role may be ascribed to the Ala118-Gly119-Alla120-Val121-Val122 turn motif that seemingly interacts with a hydrophobic pocket formed by the H2/H2-H3/H3 bundle.

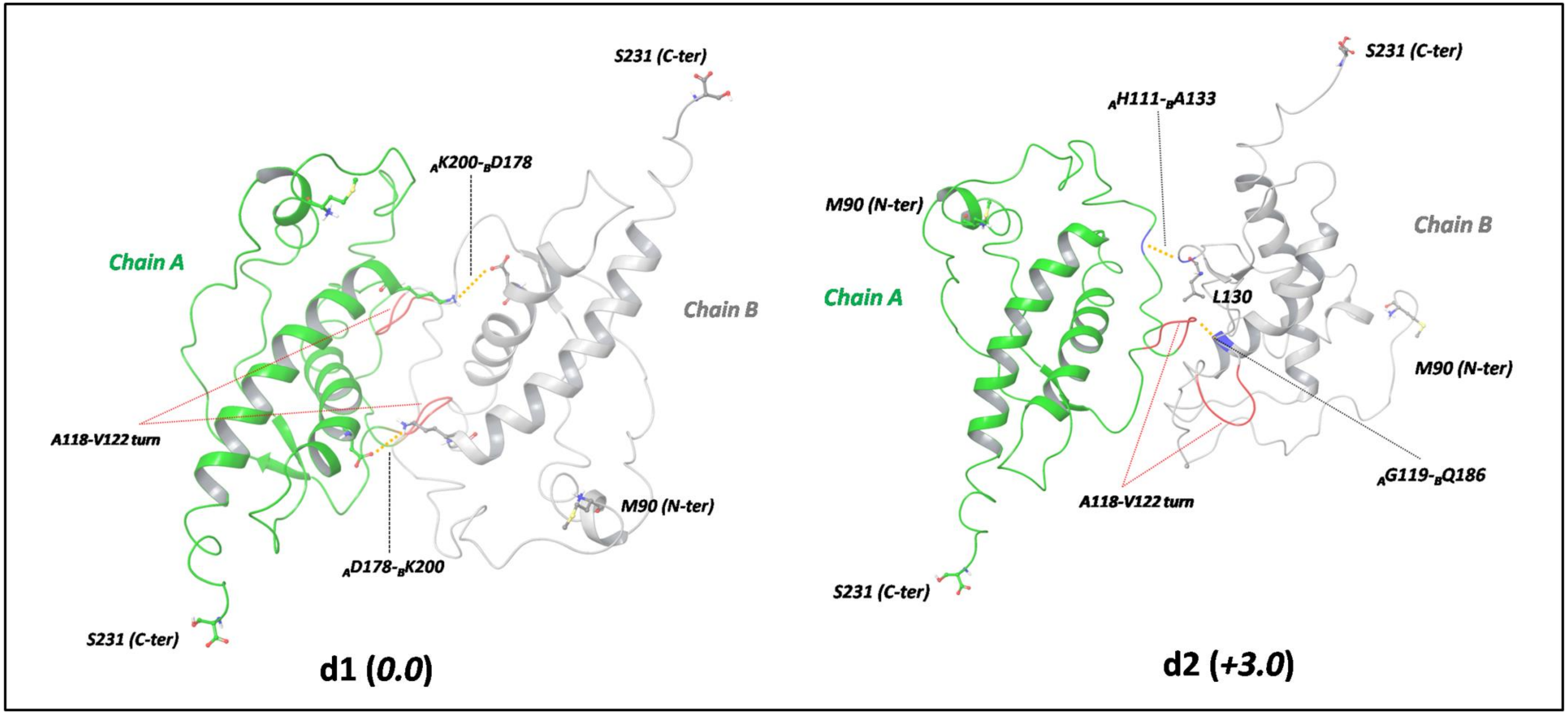

Fig. S3 The lowest energy of dimeric E200K complexes (**d1** and **d2**) obtained by performing protein-protein docking. The relative total energy scores are reported in parenthesis. The A118-V122 turn motives are red colored and H bonds are reported by using a yellow dashed line

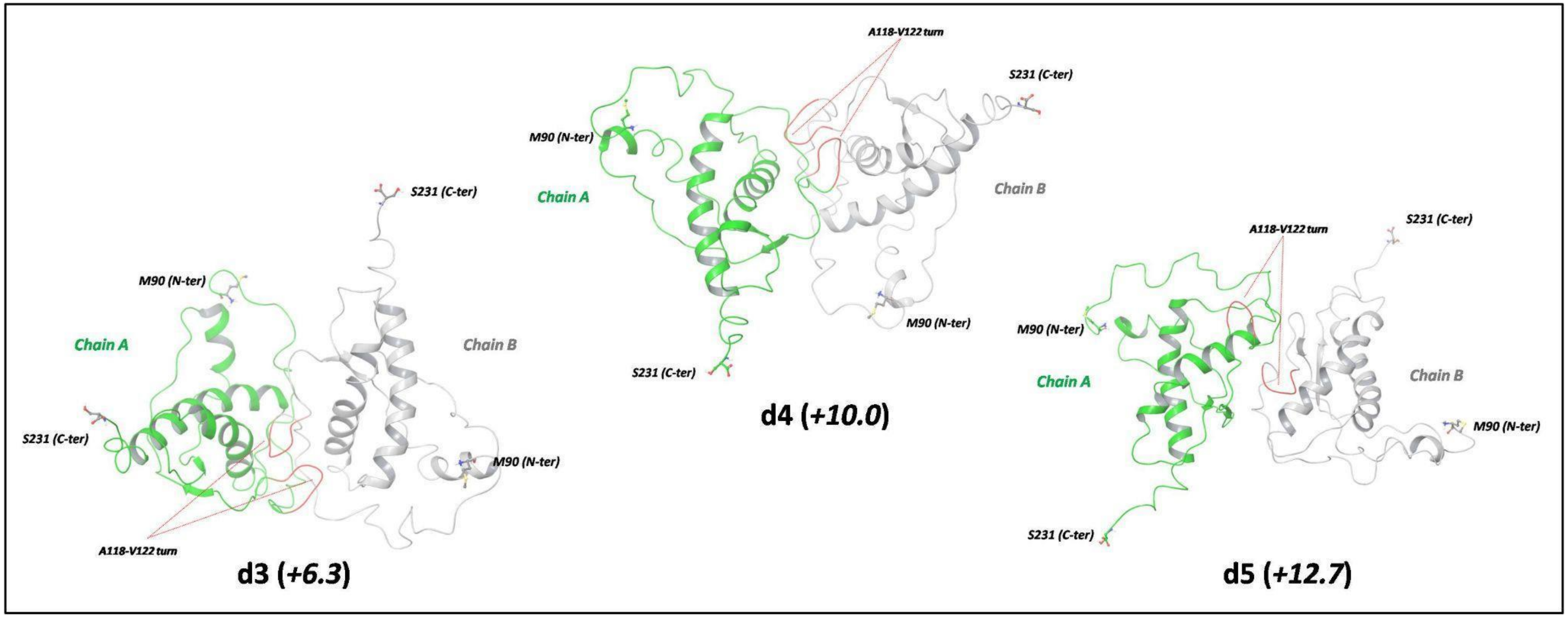

Fig. S4 The lowest energy of dimeric E200K complexes (**d3**, **d4** and **d5**) obtained by performing protein-protein docking. The relative total energy scores are reported in parenthesis. The A118-V122 turn motives are red colored

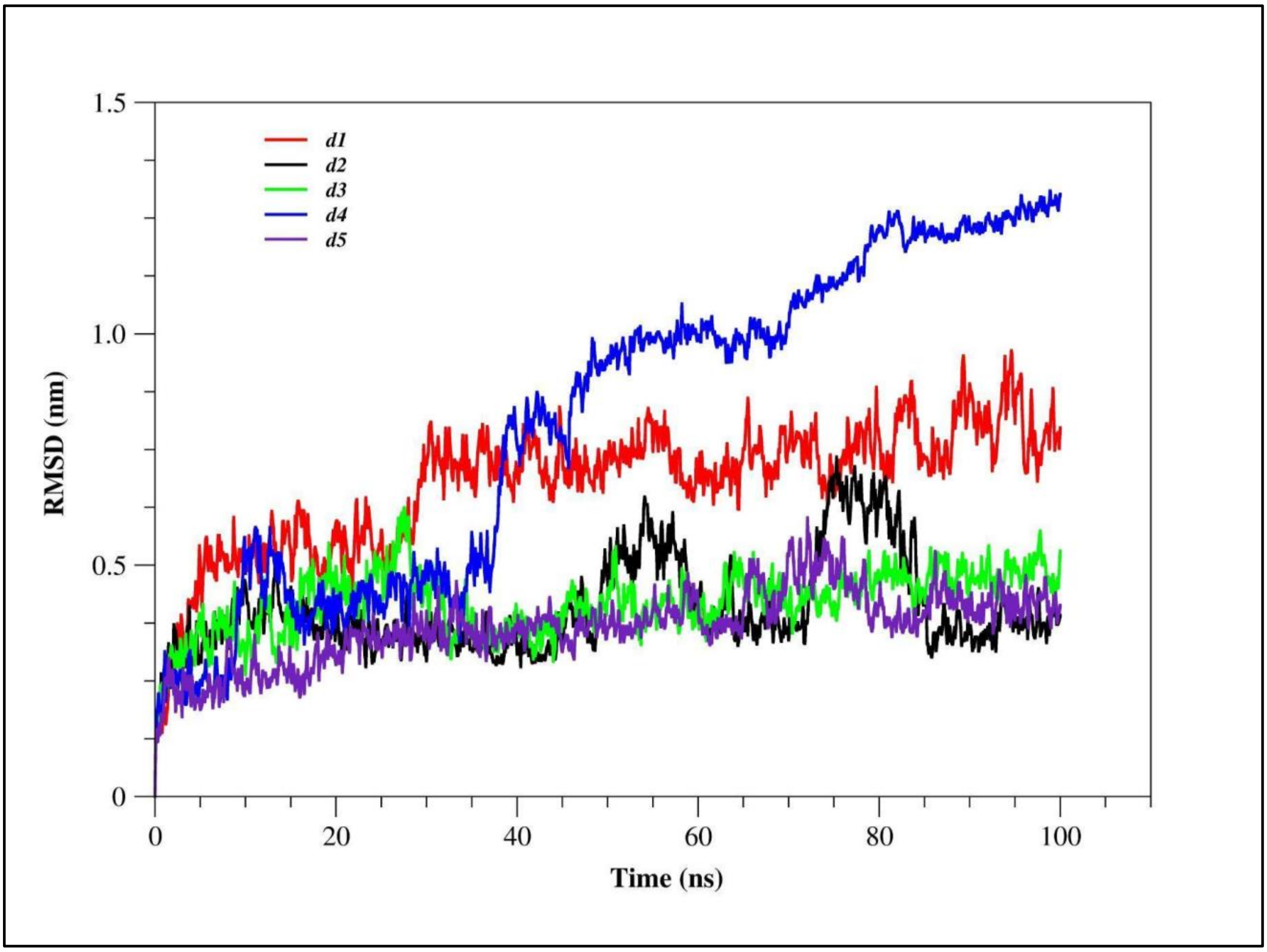

Fig. S5 The root mean squared deviation of the backbone coordinates of 90-231 PrP-E200K dimers, namely **d1**, **d2**, **d3**, **d4** and **d5**, reported in red, black, green, blue and violet line, respectively

Table S3 Results of clustering analysis of E200K dimeric complexes

E200K dimeric complex	#cluster	cluster names	weights
d1	2	d1-a, d1-b	0.92, 0.08
d2	3	d2-a, d2-b, d2-c	0.83, 0.10, 0.07
d3	2	d3-a, d3-b	0.94, 0.06
d4	3	d4-a, d4-b, d4-c	0.77, 0.16, 0.07
d5	2	d5-a, d5-b	0.89, 0.11

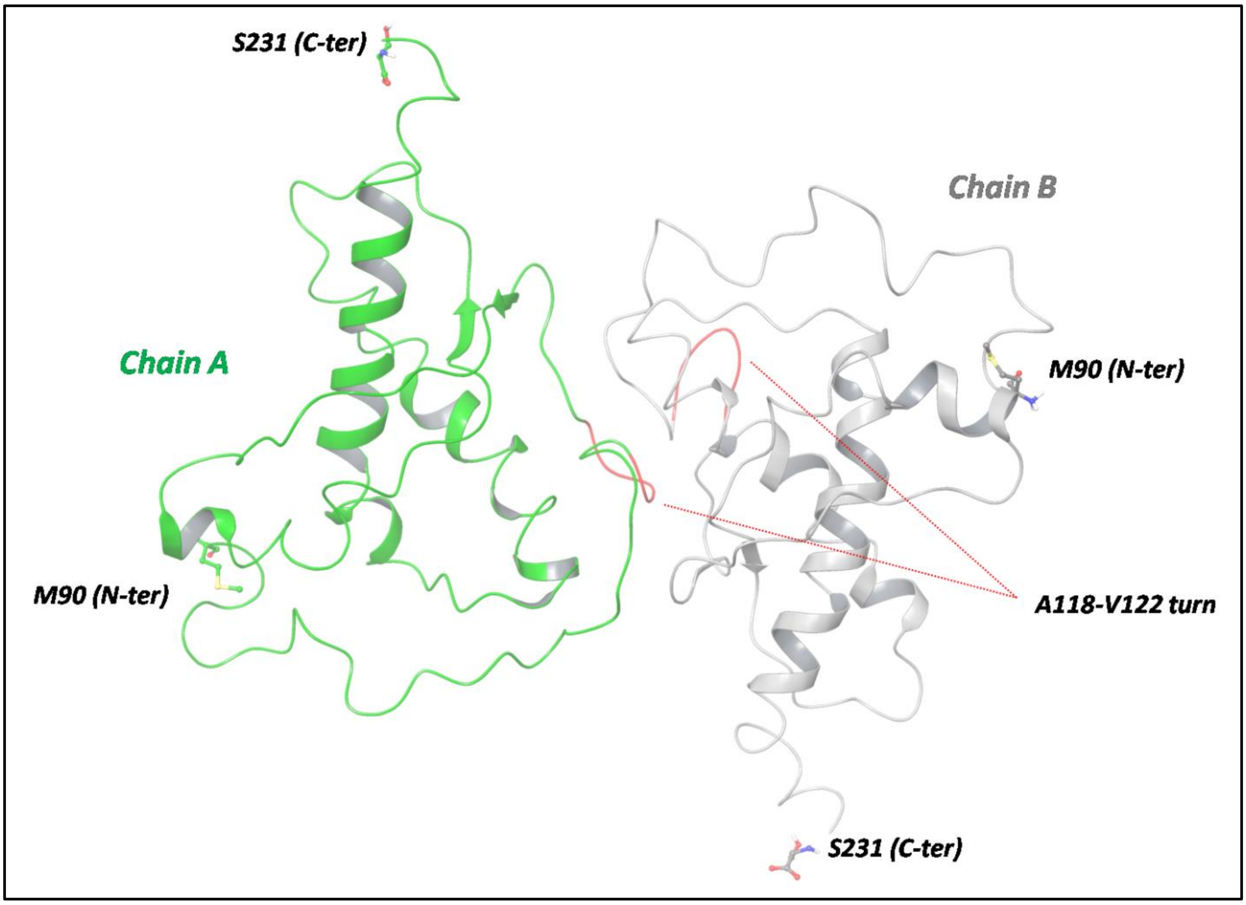

Fig. S6 Structure of **d2-a** E200K dimeric complex, the most representative structure of MD trajectory of **d2** according to cluster analysis. The A118-V122 turn motives are red colored

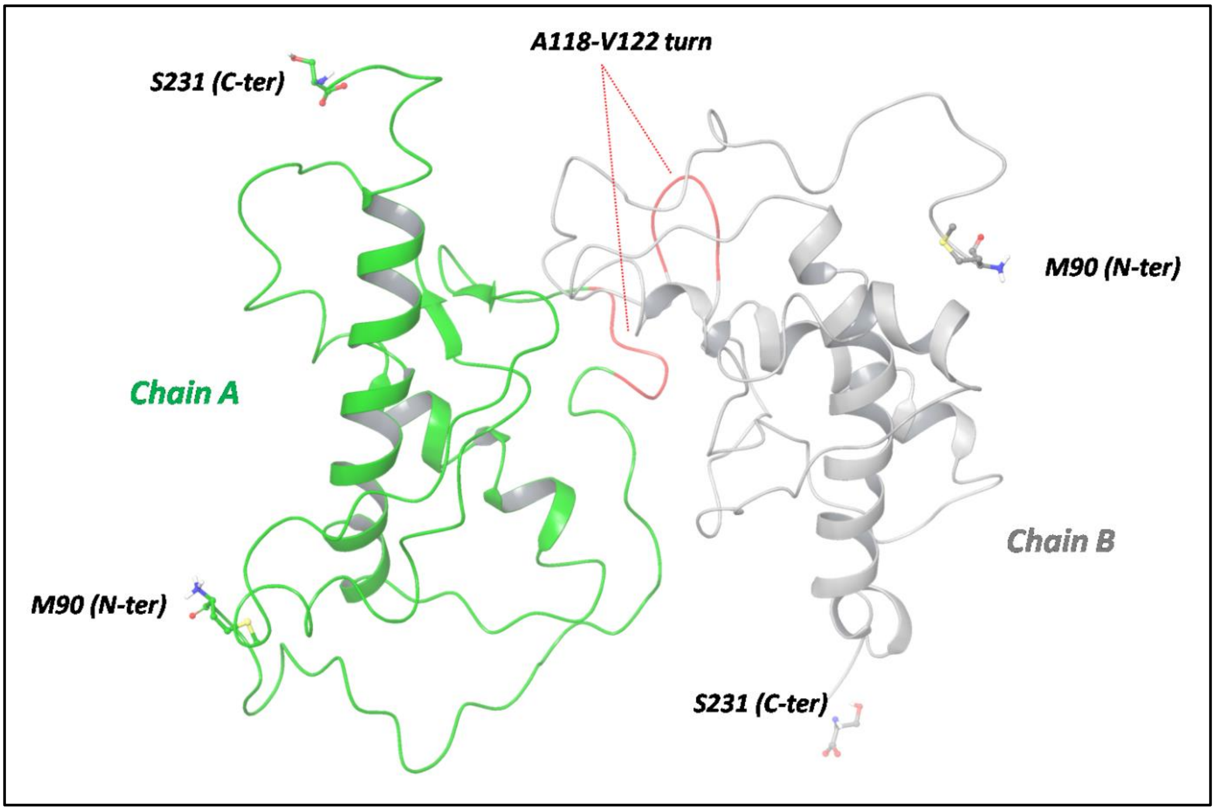

Fig. S7 Structure of **d2-b** E200K dimeric complex, resulting from cluster analysis of MD trajectory. The A118-V122 turn motives are red colored

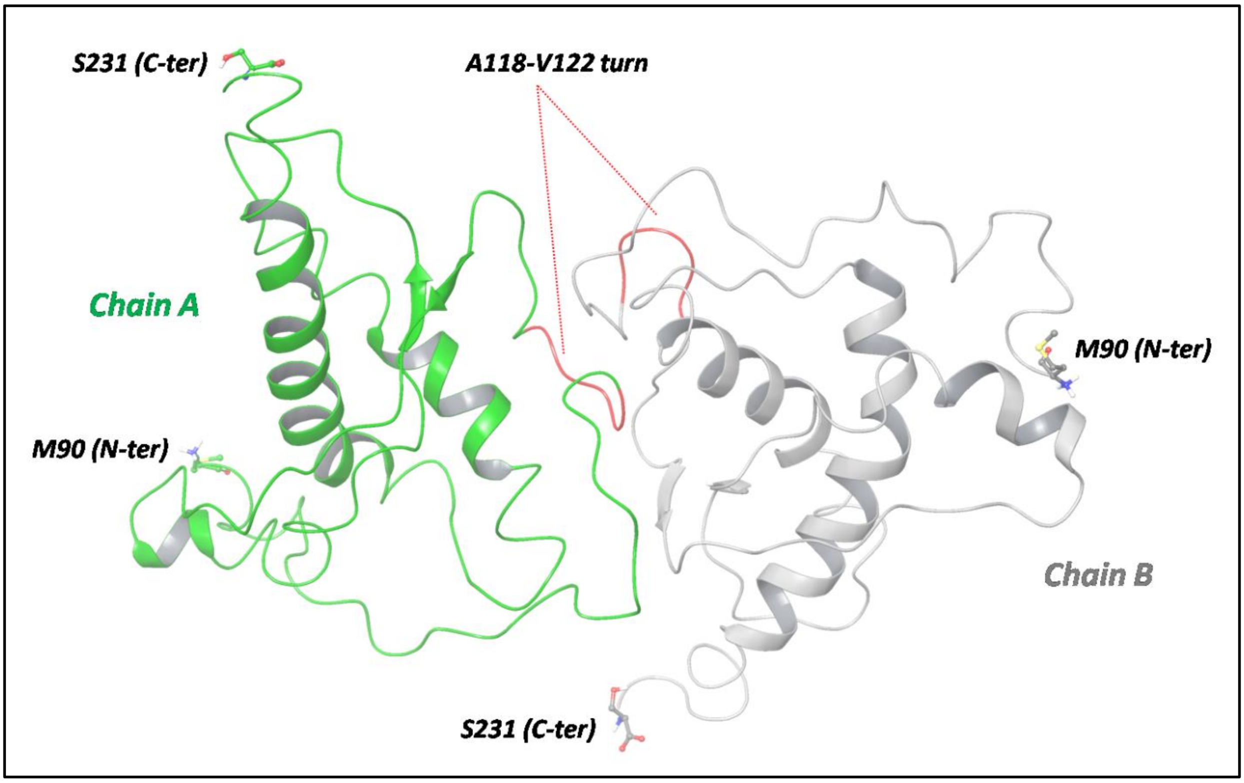

Fig. S8 Structure of **d2-c** E200K dimeric complex, resulting from cluster analysis of MD trajectory. The A118-V122 turn motives are red

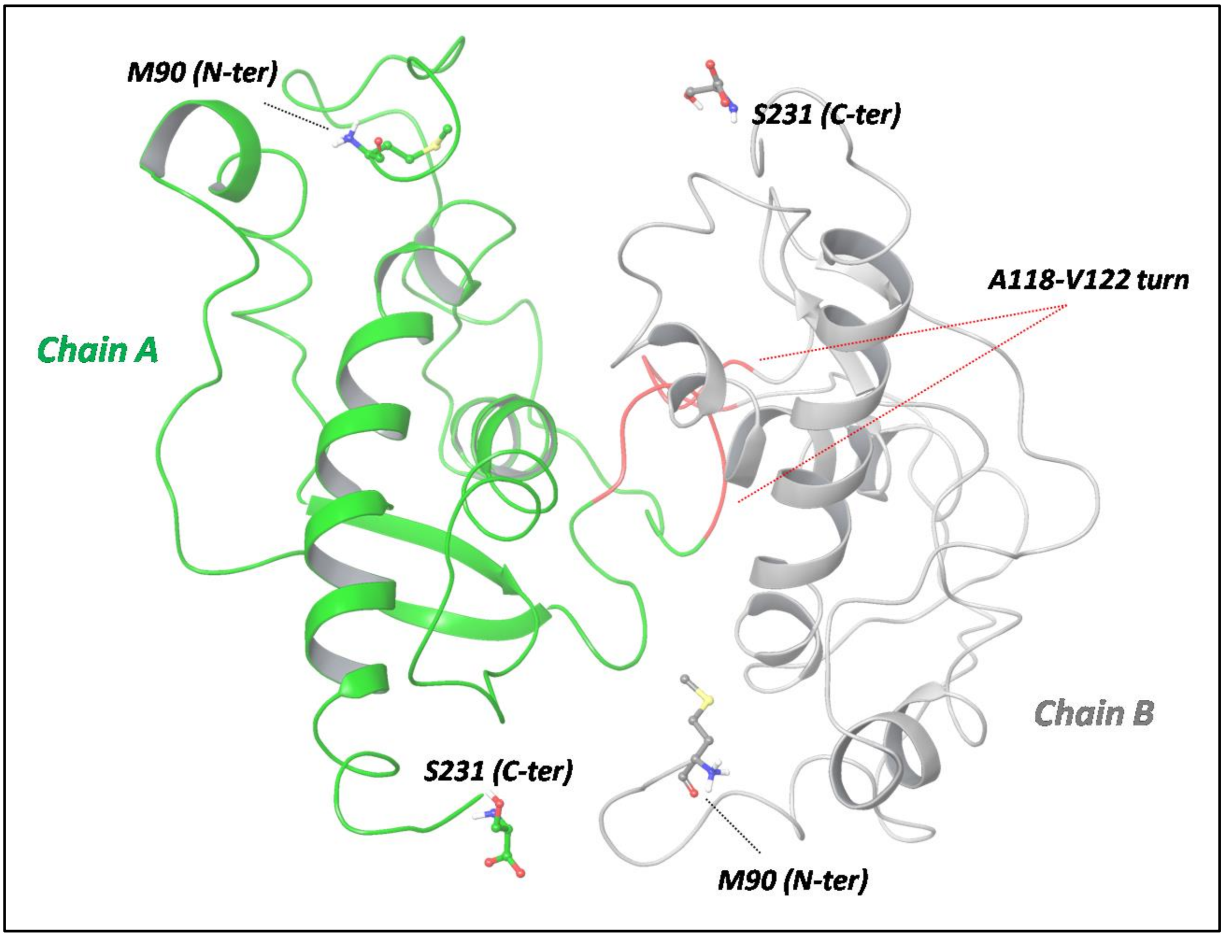

Fig. S9 Structure of **d4-a** E200K dimeric complex, the most representative structure of MD trajectory of d4 according to cluster analysis. The A118-V122 turn motives are red colored

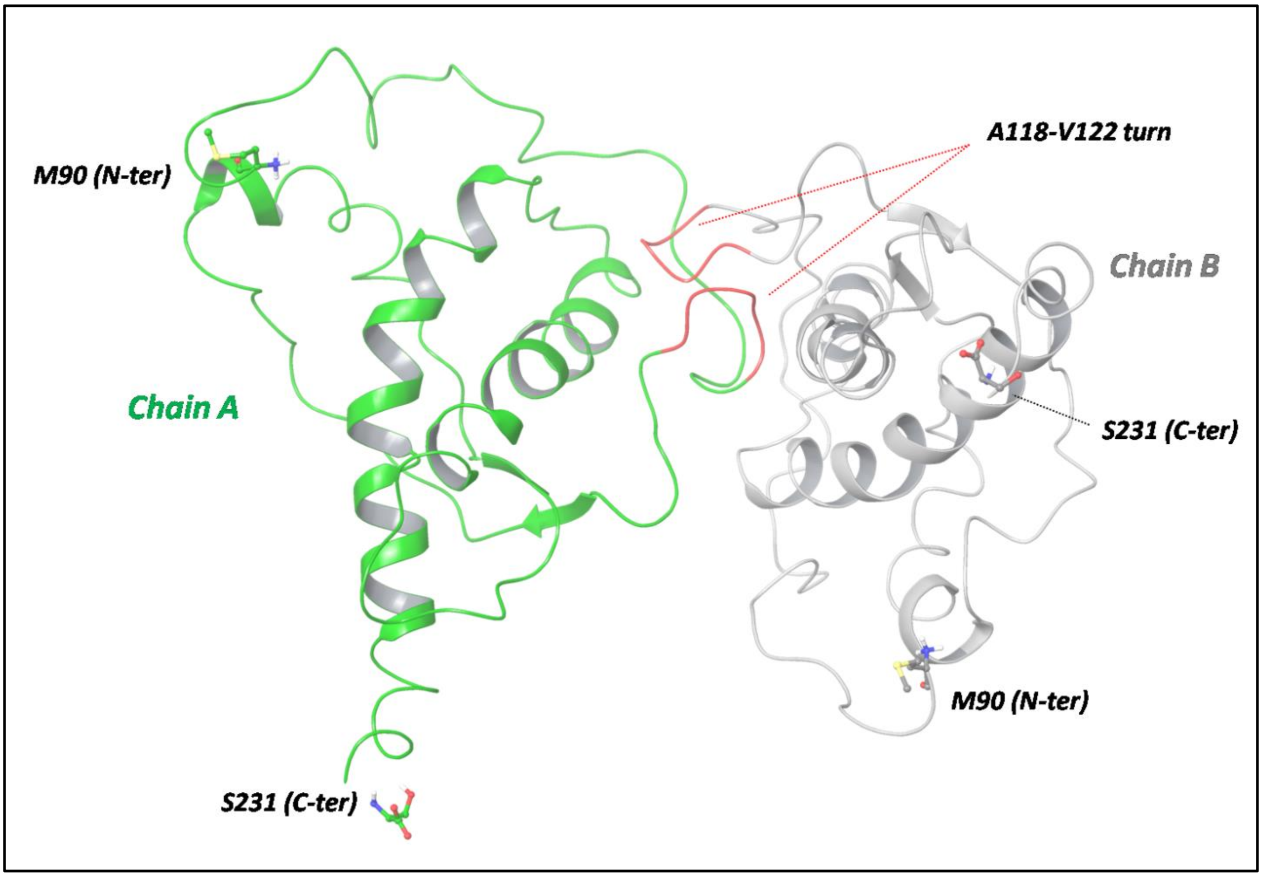

Fig. S10 Structure of **d4-b** E200K dimeric complex, resulting from cluster analysis of MD trajectory. The A118-V122 turn motives are red colored

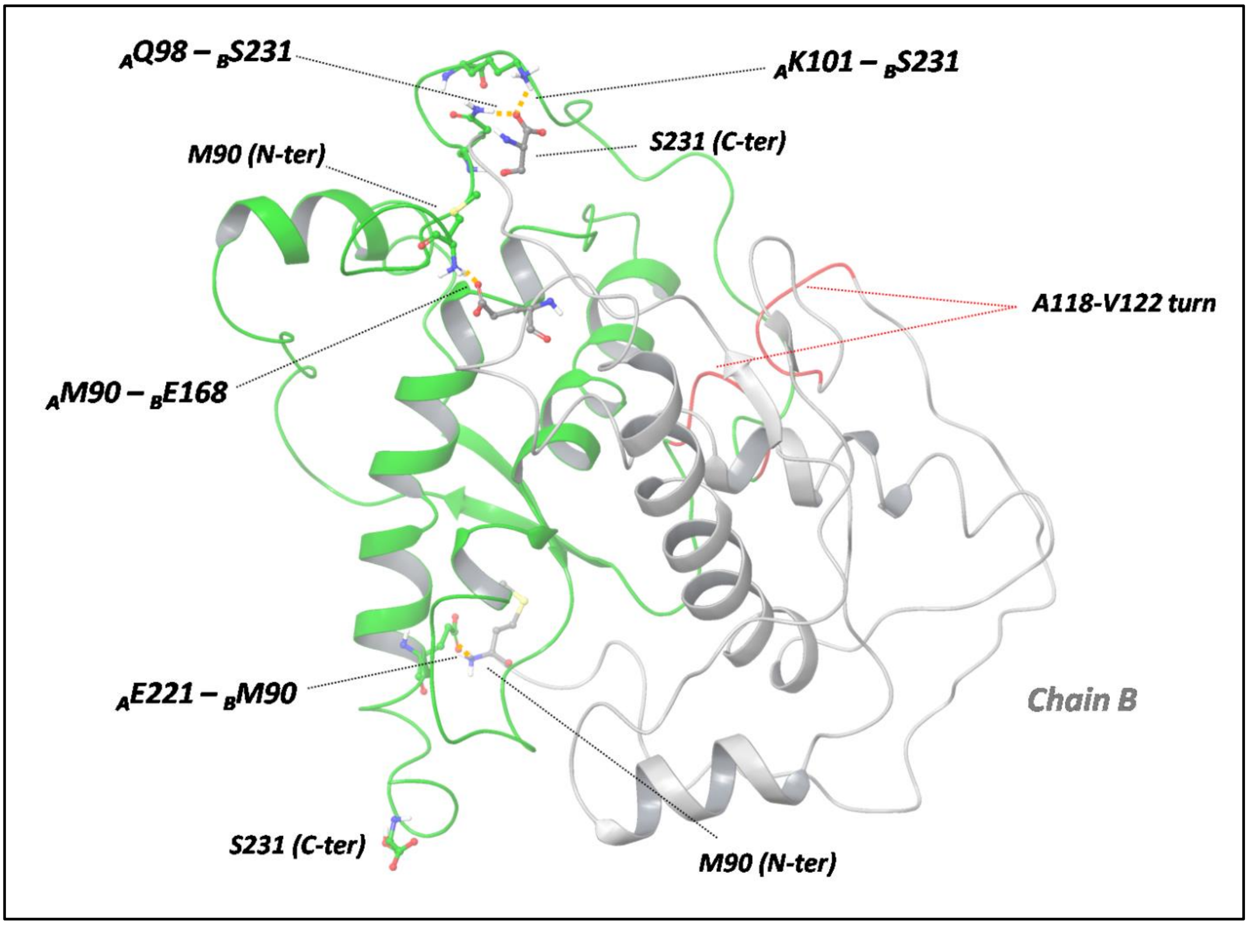

Fig. S11 Structure of **d4-c** E200K dimeric complex, resulting from cluster analysis of MD trajectory. The A118-V122 turn motives are red colored and H bonds are reported by using yellow dashed line

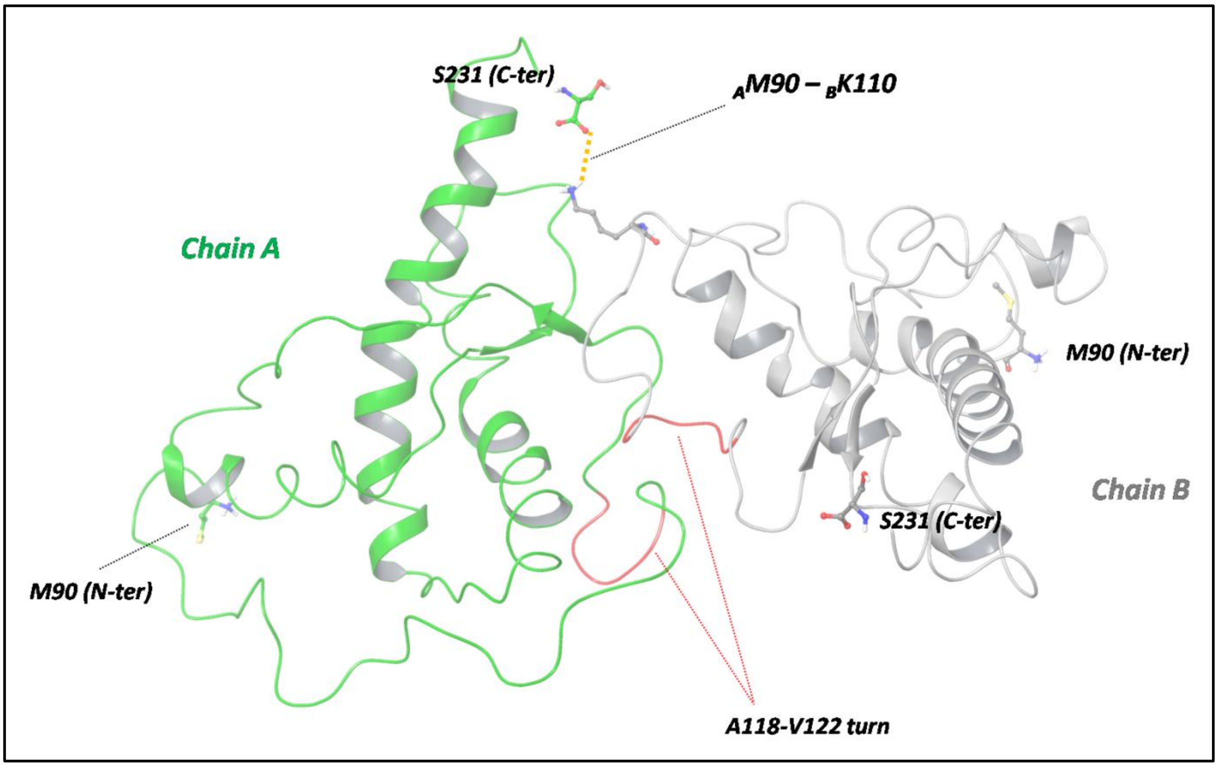

Fig. S12 Structure of **d5-a** E200K dimeric complex, the most representative structure of MD trajectory of **d5** according to cluster analysis. The A118-V122 turn motives are red colored and H bonds are reported by using a yellow dashed line

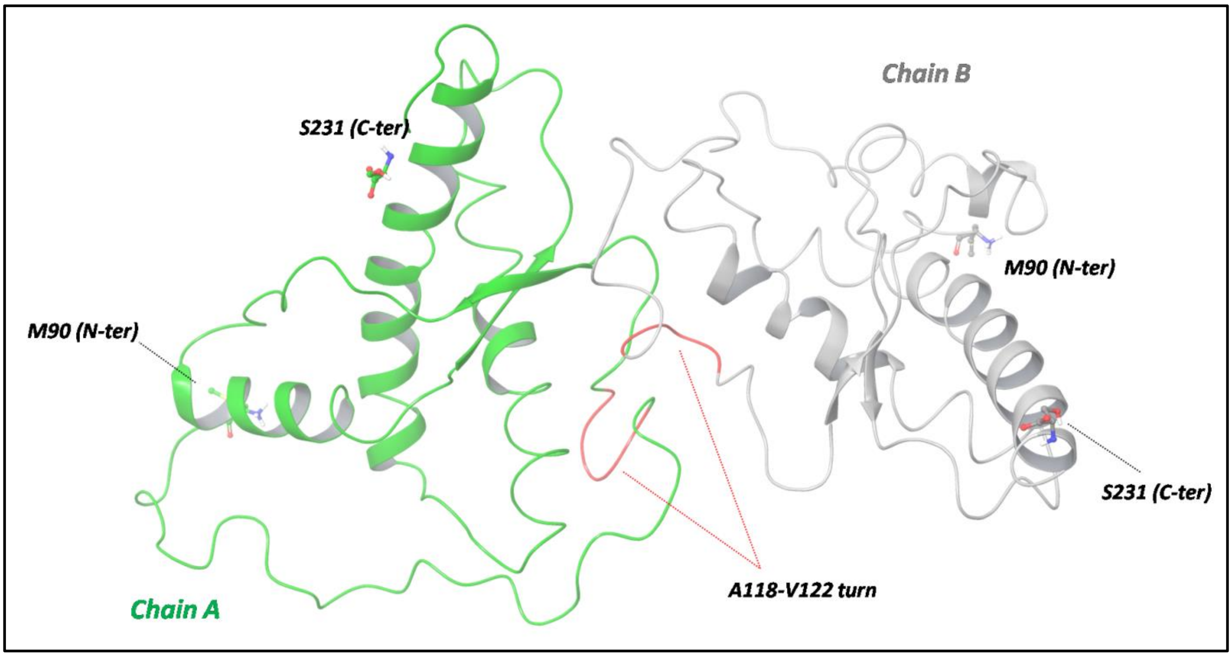

Fig. S13 Structure of **d5-b** E200K dimeric complex, resulting from cluster analysis of MD trajectory. The A118-V122 turn motives are red colored

Table S4 Binding energies, ΔE^{FMO}, and pair interaction energies between residues of the two chains A and B, PIE^{AB}, of the most representative structures resulting from the clustering analysis of E200K complexes

**E200K dimeric complexes**	**ΔE^{FMO} (kcal/mol)**	**PIE^{AB} (kcal/mol)**
d1-a	29.8	-71.9
d1-b	-240.3	-65.9
d2-a	44.7	-102.5
d2-b	-26.2	-230.6
d2-c	-155.8	-210.0
d3-a	10.1	-220.0
d3-b	60.7	-96.7
d4-a	187.7	-125.4
d4-b	91.4	-92.0
d4-c	-151.3	-828.2
d5-a	44.8	-96.7
d5-b	-91.6	-140.7

.

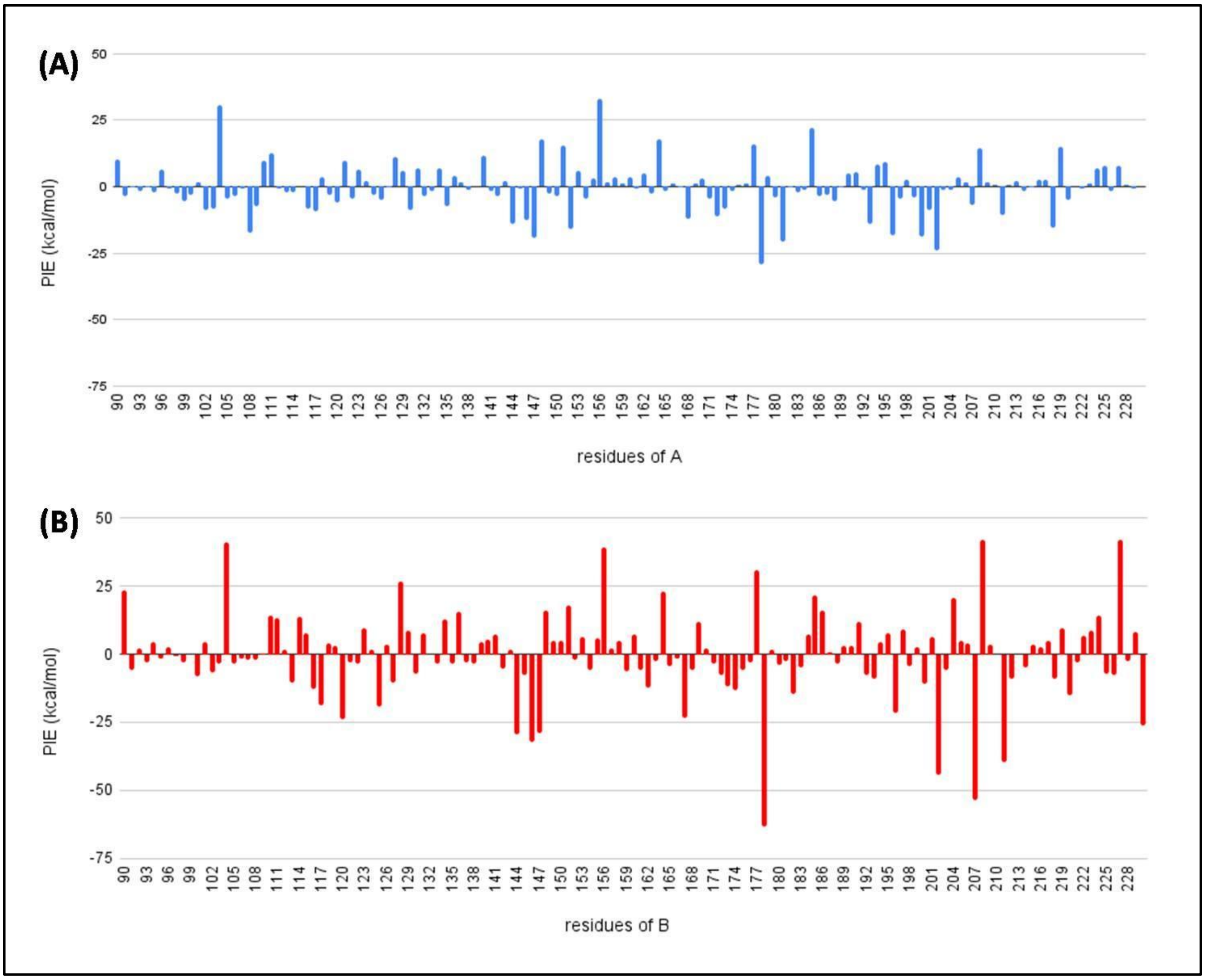

Fig. S14 Weighted PIE values computed for **d1**, between residues of chain A and the whole chain B (blue bar) and vice versa (red bar). The most attractive interactions were assigned to Asp178 both in chain A (-30 kcal/mol) and chain B (-63 kcal/mol): the $_{\mathrm{A}}$Asp178 establishes an attractive interaction with $_{\mathrm{B}}$Arg208 (-8 kcal/mol) while $_{\mathrm{B}}$Asp178 strongly interacts with $_{\mathrm{A}}$Lys200 (-19 kcal/mol), in agreement with the geometrical features discussed above. Another important residue is $_{\mathrm{B}}$Asp167, which participates in the attractive interaction with $_{\mathrm{A}}$Lys104 (-10 kcal/mol), and other residues contributing to the stabilization of **d1** are Asp144, Glu146, Glu168, Asp202, Glu207 and Glu211, again, displaying more negative per-residue PIE in the chain B residues

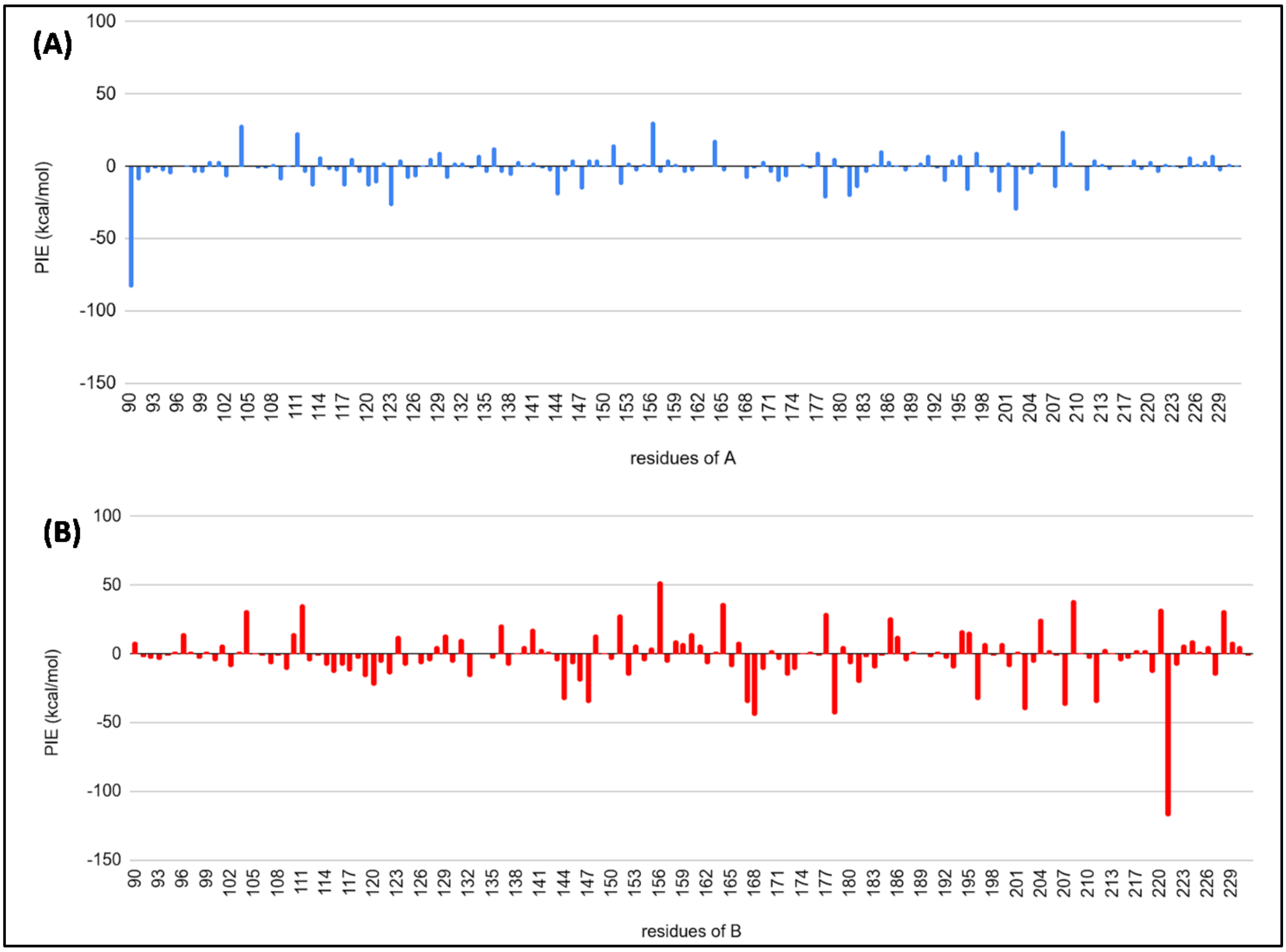

Fig. S15 Weighted PIE values computed for **d3**, between residues of chain A and the whole chain B (blue bar) and vice versa (red bar)

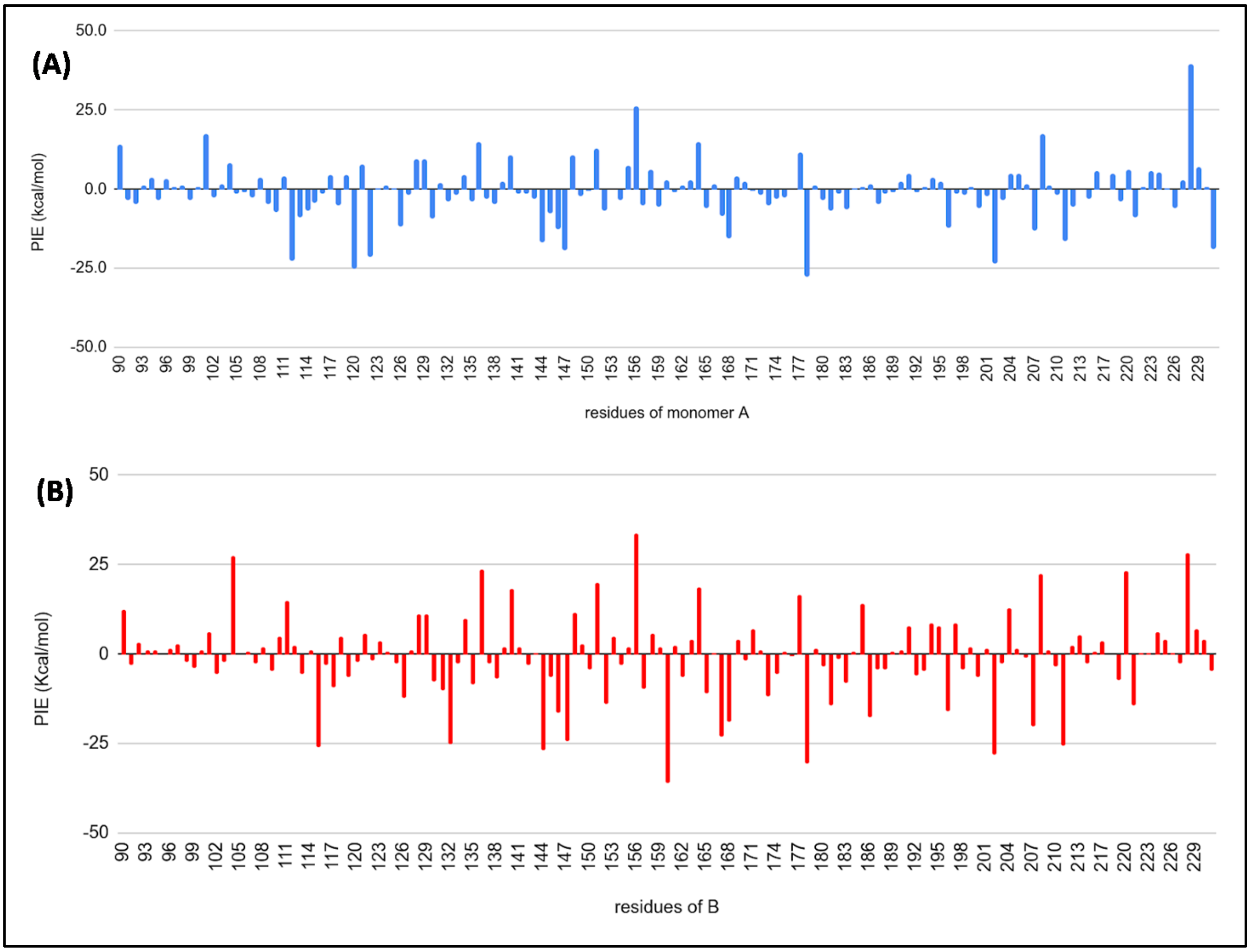

Fig. S16 Weighted PIE values computed for **d2**, between residues of chain A and the whole chain B (blue bar) and vice versa (red bar)

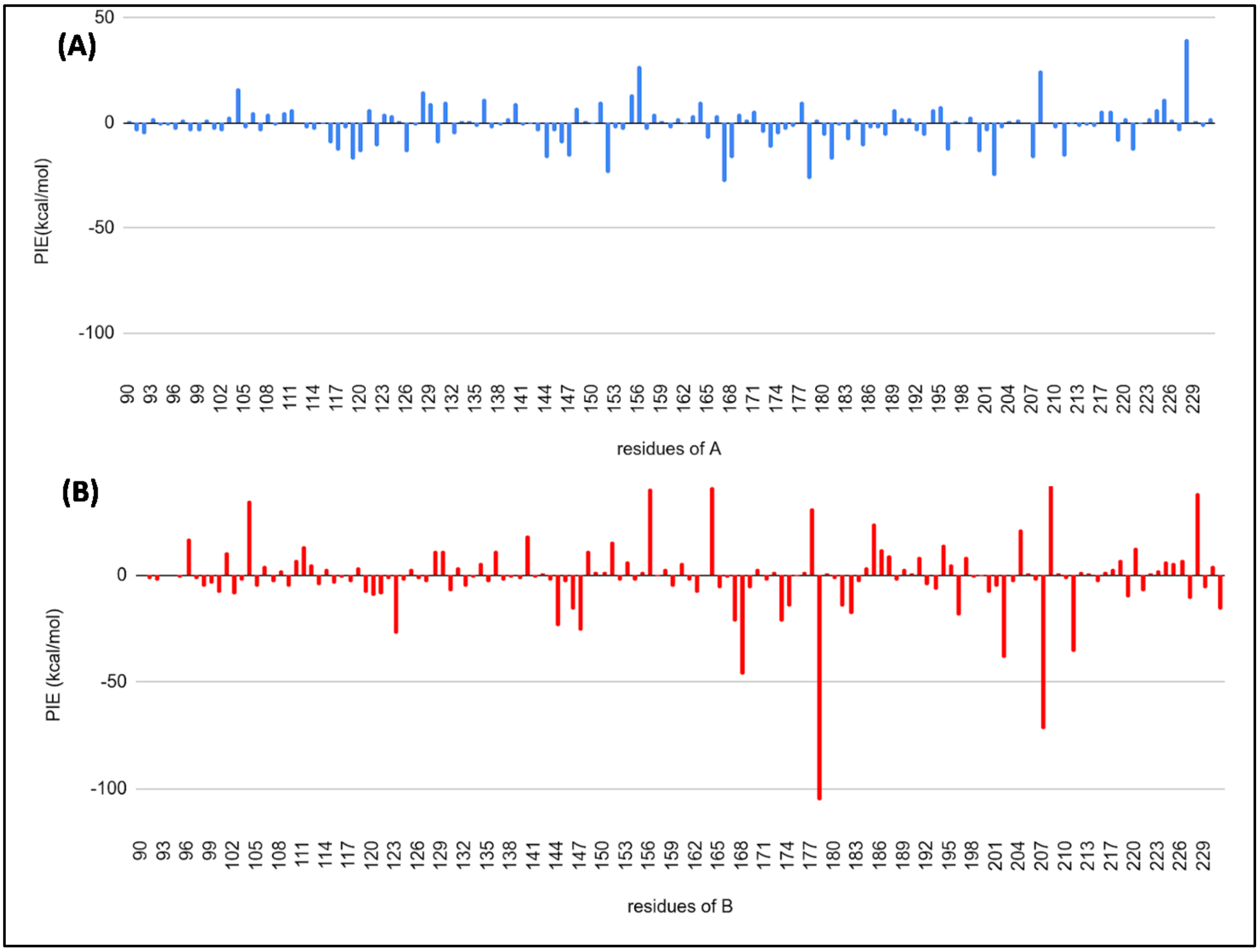

Fig. S17 Weighted PIE values computed for **d4**, between residues of chain A and the whole chain B (blue bar) and vice versa (red bar)

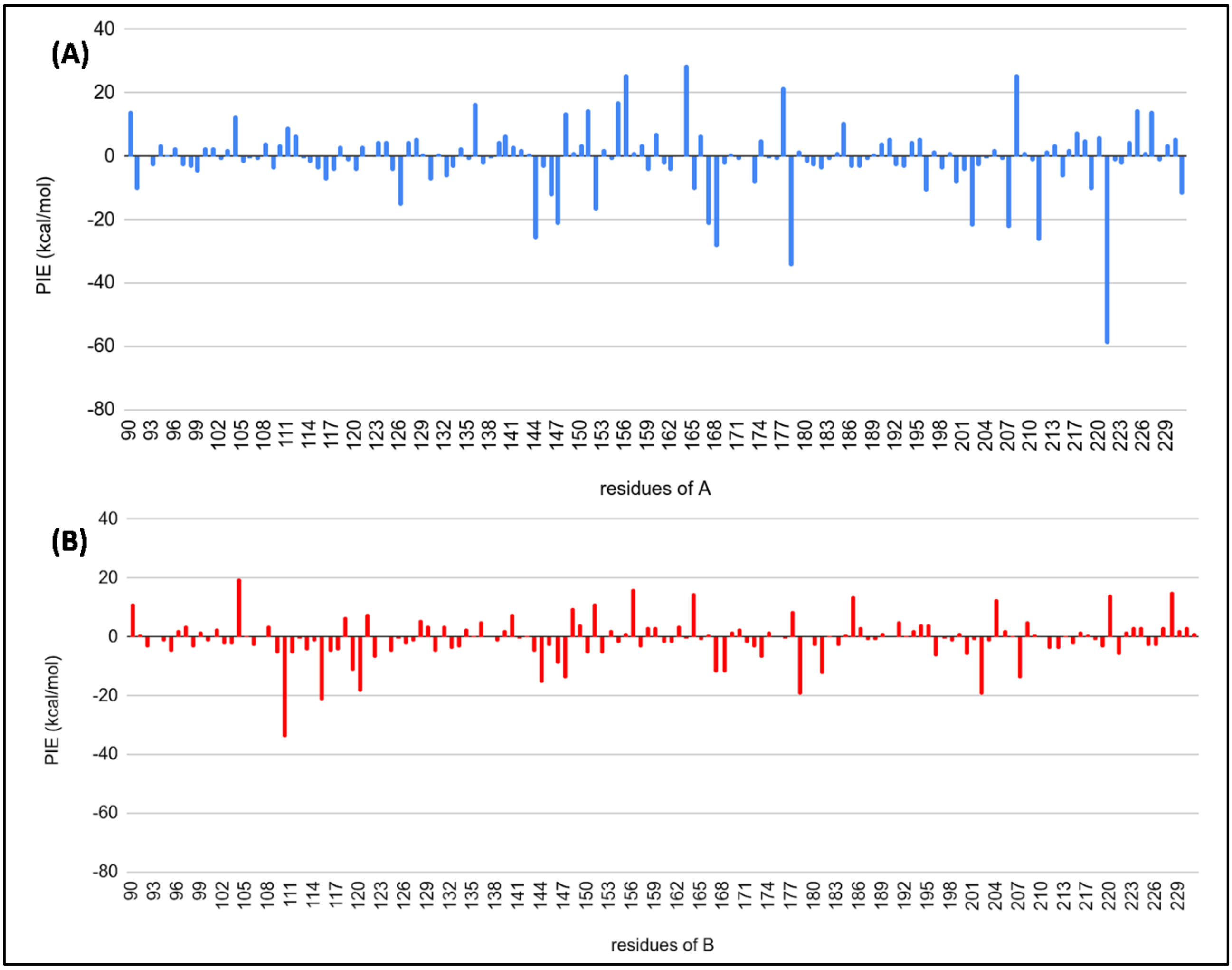

Fig. S18 Weighted PIE values computed for **d5**, between residues of chain A and the whole chain B (blue bar) and vice versa (red bar)

Table S5 weighted PIE values (in kcal/mol) between secondary structure domains of chain A with the whole protein B. PIEs are referred to **d1-d5** complexes

Protein domain of subunit A interacting with whole subunit B	weighted PIE - homodimeric complexes of 90-231 PrP-E200K				
	**d1**	**d2**	**d3**	**d4**	**d5**
**N-ter**	-6.8	-56.4	-157.0	-50.0	-26.7
**S1**	-2.0	0.3	1.4	0.0	-7.6
**S1-H1**	16.7	11.1	12.0	18.1	8.8
**H1**	-36.2	-42.8	-20.4	-53.0	-24.6
**H1-S2**	64.1	26.7	25.8	36.4	26.7
**S2**	6.3	3.9	0.2	4.0	-4.6
**S2-H2**	-34.9	-17.4	-13.8	-48.4	-21.1
**H2**	-26.6	-40.8	-33.6	-67.6	-21.1
**H2-H3**	-32.8	-8.2	-17.2	-13.6	-11.1
**H3**	-46.6	-24.9	-30.7	-45.7	-72.4
**C-ter**	32.9	25.5	20.9	50.6	22.0

Table S6 weighted PIE values (in kcal/mol) between secondary structure domains of chain B with the whole protein A. PIEs are referred to **d1-d5** complexes

Protein domain of subunit B interacting with whole subunit A	weighted PIE - homodimeric complexes of 90-231 PrP-E200K				
	**d1**	**d2**	**d3**	**d4**	**d5**
**N-ter**	8.6	16.0	-46.7	-8.8	-87.1
**S1**	7.4	3.2	8.5	4.6	-1.1
**S1-H1**	11.1	-3.6	27.4	23.2	7.8
**H1**	-52.0	-58.0	-72.0	-41.8	-27.5
**H1-S2**	55.5	2.3	93.8	47.0	5.3
**S2**	-11.1	-2.5	-6.6	-7.5	-15.3
**S2-H2**	-75.8	-41.7	-85.2	-77.0	-15.0
**H2**	-16.1	-49.8	-11.6	-59.5	-11.9
**H2-H3**	-8.2	-2.0	-10.2	-1.7	3.9
**H3**	-50.4	-23.3	-146.6	-71.1	-10.5
**C-ter**	65.2	36.6	36.6	23.4	19.5

Table S7 Weighted relative intra-domain interaction energies,ΔPIE, of H1, H2 and H3 computed between E200K and *chains A* of E200K-dimers. In details, H3 domain presented the highest ΔPIE values of +183.7 (chain A of **d5**), +179.6 (chain A **d2**), +162.4 (chain A of **d1**), +151.1 (chain B of **d5**) and +125.4 (chain B of **d1**) kcal/mol

**Domain - chain A**	**Intra-domain ΔPIE (kcal/mol)**				
	**d1**	**d2**	**d3**	**d4**	**d5**
H1	-14.9	+64.1	-5.1	+60.0	+2.2
H2	+32.4	+40.7	+77.7	+11.3	+57.9
H3	+162.4	+179.6	+149.3	+72.6	+183.7

Table S8 Weighted relative intra-domain interaction energies, ΔPIE, of H1, H2 and H3 computed between E200K and *chains B* of E200K-dimers

**Domain- Chain B**	**Intra-domain ΔPIE (kcal/mol)**				
	**d1**	**d2**	**d3**	**d4**	**d5**
H1	-2.5	+44.2	+21.7	-21.7	+12.8
H2	+59.1	+55.2	+59.1	+18.5	+46.0
H3	+125.4	+94.2	+42.9	+21.4	+151.1

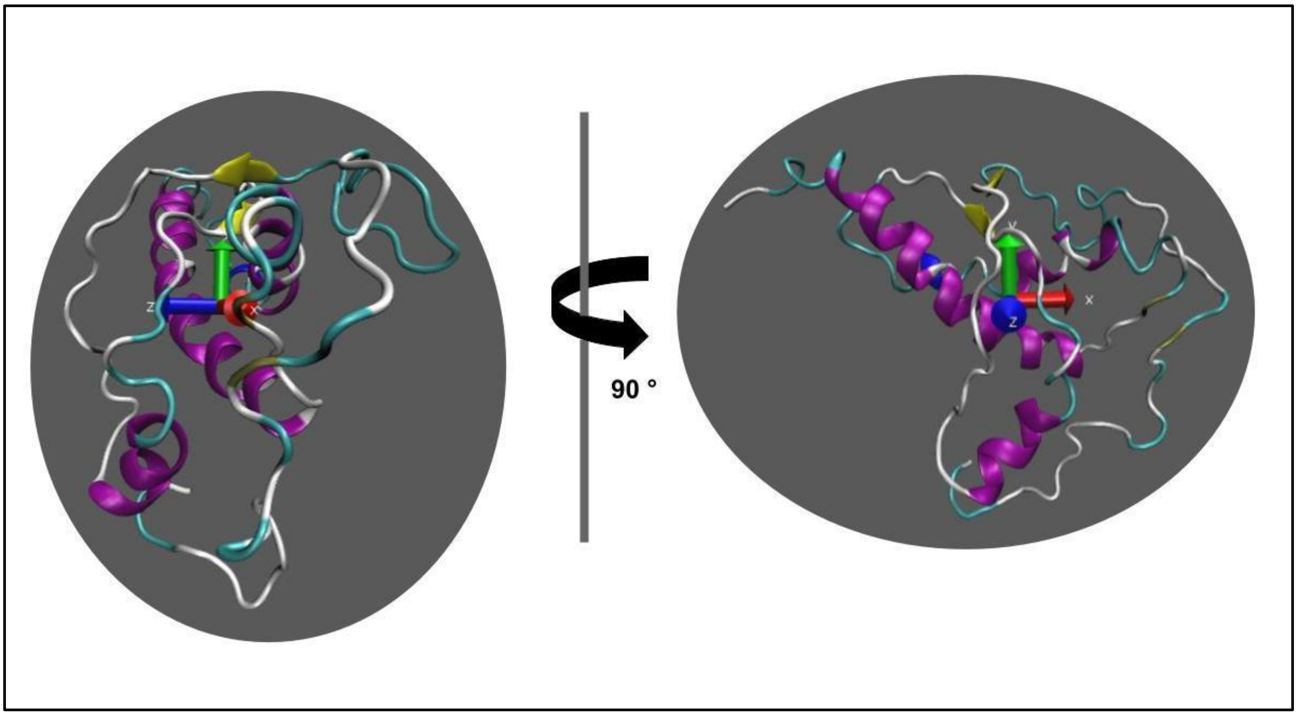

Fig. S19 position of xyz origin with respect to 90-231 PrP-E200K structure (coordinates) used for MEP and DRY MIF calculations (ATOMIF)

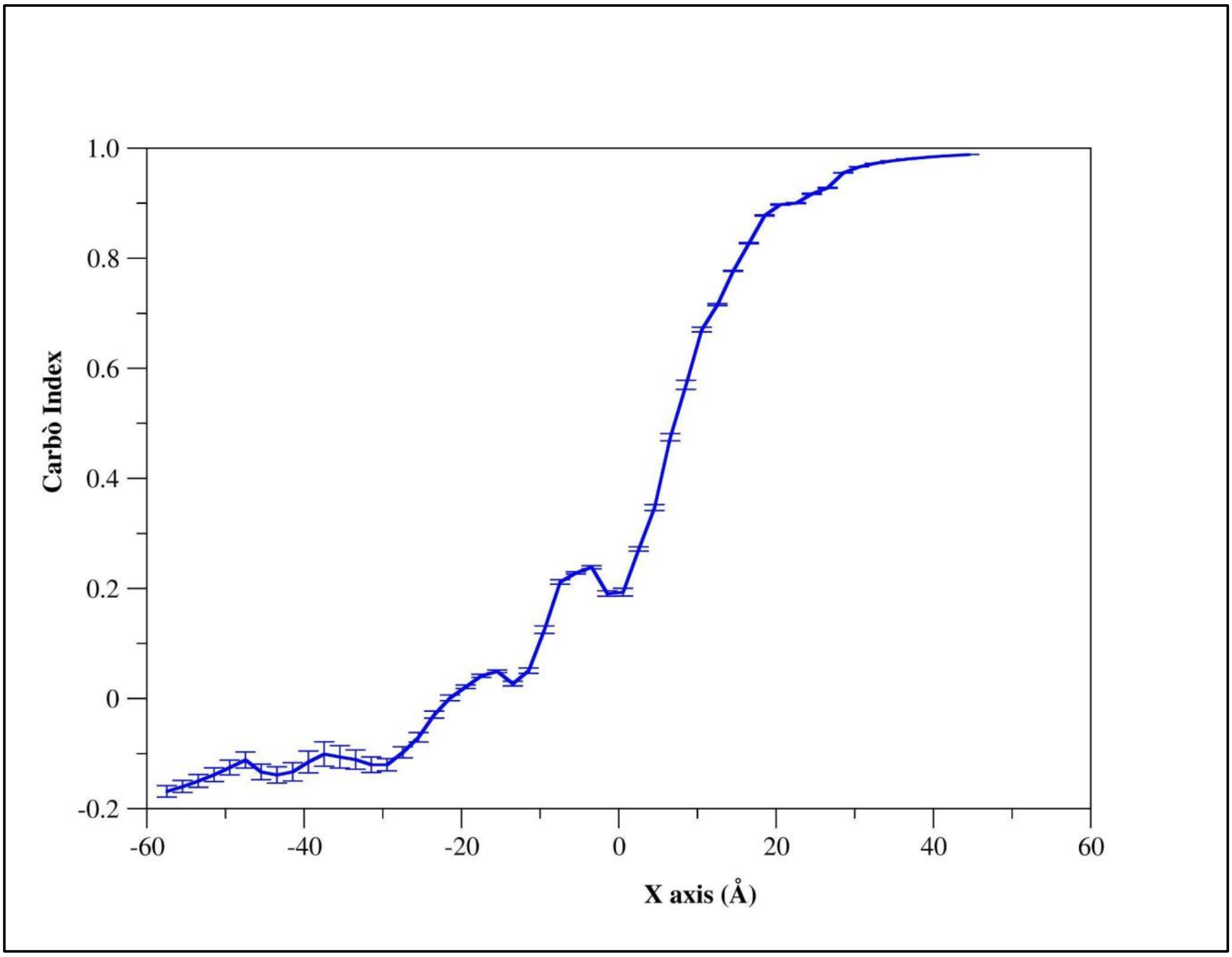

Fig. S20 Weighted Carbo MEP cross-similarity profile of the 90-231 and 120-231 PrP-E200K segments, computed along the x axis (see Fig. S19 as reference). The weighted standard deviation is also reported

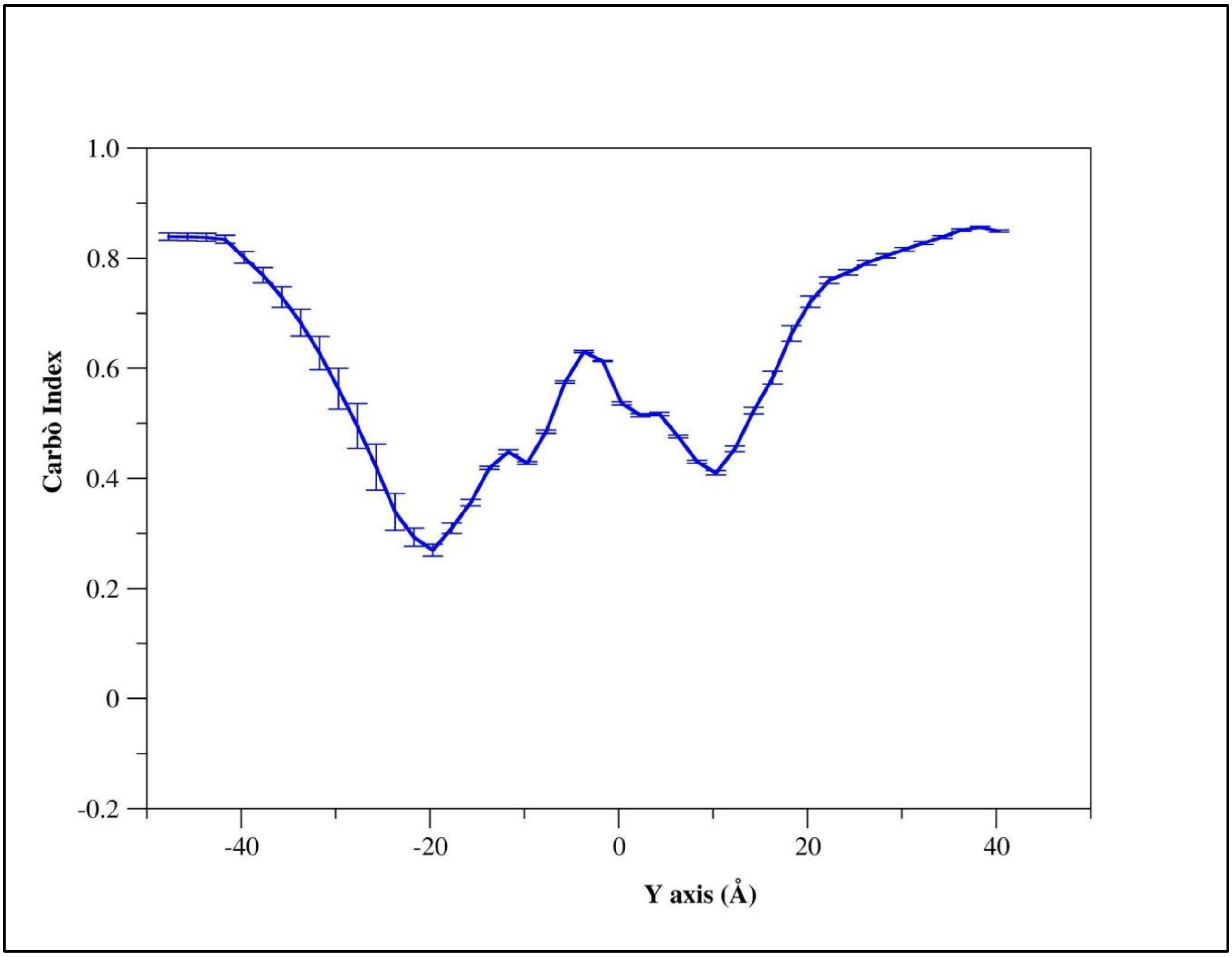

Fig. S21 Weighted Carbo MEP cross-similarity profile of the 90-231 and 120-231 PrP-E200K segments, computed along the y axis (see Fig. S19 as reference). The weighted standard deviation is also reported

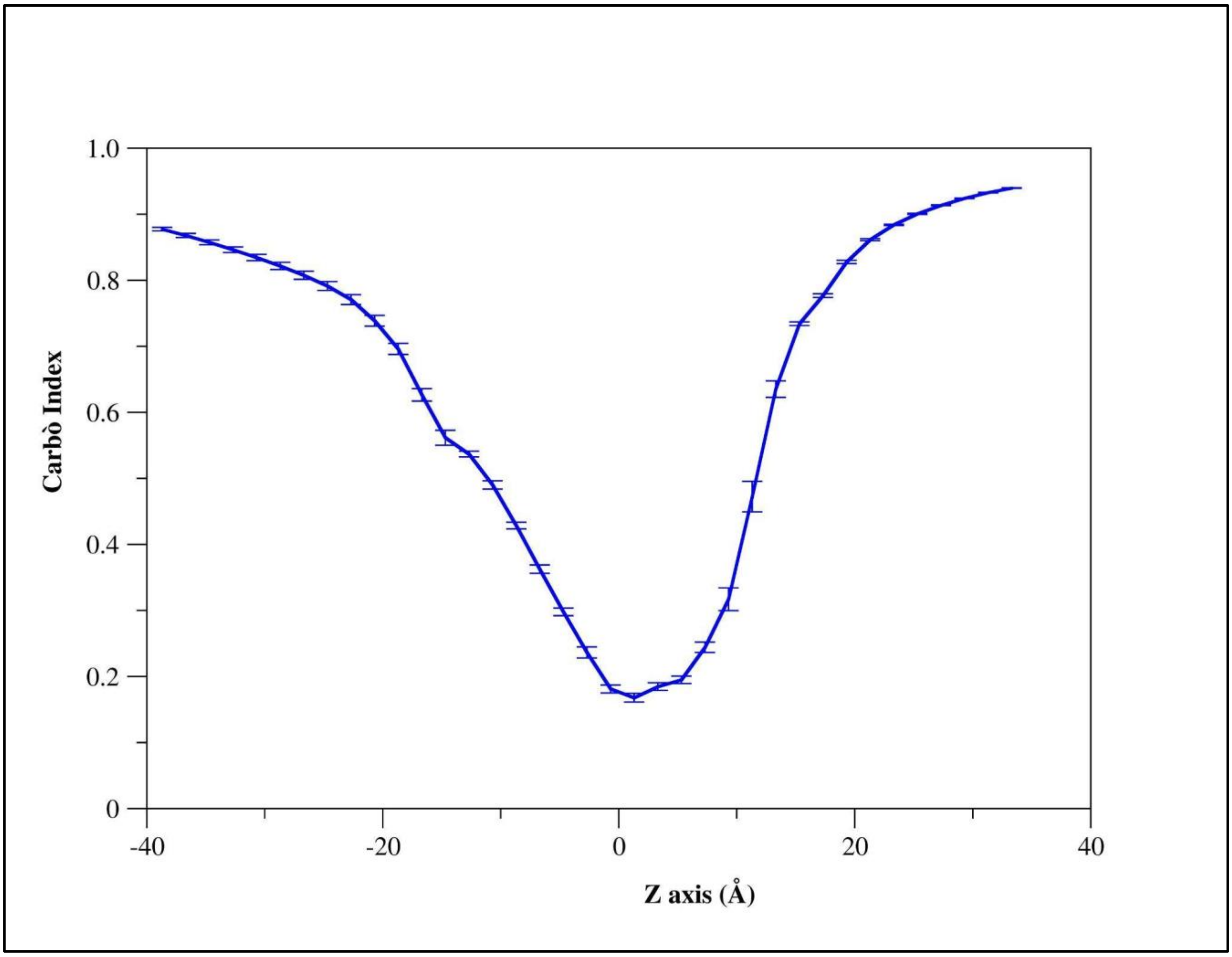

Fig. S22 Weighted Carbo MEP cross-similarity profile of the 90-231 and 120-231 PrP-E200K segments, computed along the z axis (see Fig. S19 as reference). The weighted standard deviation is also reported

Note S3 *DRY MIF and hydrophobic contacts analysis – additional details*

In the **d1** dimer, we identified two regions on chain **A** surface involved in relevant hydrophobic interactions: one is formed by the C-terminus of H2, the N-terminus of H3, and part of the H2-H3 loop, in which some residues strongly interact with hydrophobic field of chain B, in particular, Thr192(-3.2 kcal/mol), Asn197 (-2.9 kcal/mol), Thr119 (-1.1 kcal/mol) and Thr201 (-1.0 kcal/mol). The second region is located on the 90-120 fragment where the most important hydrophobic residues are Pro105 (-1.0 kcal/mol), Met112 (-1.0 kcal/mol) and Ala120 (-1.0 kcal/mol). Conversely, the residues of chain B interacting with the hydrophobic field produced by subunit A, are located predominantly in the 90-125 fragment such as Ala113 (-0.9 kcal/mol), Ala115 (-1.5 kcal/mol), Ala116 (-1.9 kcal/mol), Ala120 (-4.1 kcal/mol), Gly124 (-5.0 kcal/mol), Gly127 (-1.0 kcal/mol) and Leu125 (-0.9 kcal/mol).The **d2-d4** complexes are also characterized by hydrophobic interactions with these tiled protein regions, although with an opposite location, hence the His111-Leu125 of A interacts with the envelope of H2, H2-H3, and H3 domains of B. On the contrary, in **d5** the most relevant hydrophobic contacts in **d5** involve only residues of the turn His111-> Leu125, and in particular, $_{A}$Gly124 (-1.3 kcal/mol), $_{A}$Leu130 (-3.4 kcal/mol), $_{A}$Met129 (-5.0 kcal/mol), $_{A}$Pro165 (-11.4 kcal/mol) of chain A with $_{B}$Ala115 (-2.6 kcal/mol), $_{B}$Ala120 (-4.7 kcal/mol), $_{B}$Lys110 (-6.1 kcal/mol) and $_{B}$Met112 (-4.7 kcal/mol) of chain B.

Table S9 Hydrophobic interaction energies, in kcal/mol, of residues involved in the most significant hydrophobic contacts (hydrophobic energies ≤ -0.9 kcal/mol)

Structure	*Per-residues hydrophobic interaction energy (kcal/mol)	
	**Chain A residues vs DRY MIF of B**	**Chain B residues vs DRY MIF of A**
**d1**	Ala120 (-1.0), Asn197 (-2.9), Met112 (-1.0), Pro105 (-1.0), Thr192 (-3.2), Thr199 (-1.1), Thr201 (-1.0)	Ala113 (-0.9), Ala115 (-1.5), Ala116 (-1.9), Ala120 (-4.1), Gly124 (-5.0), Gly127 (-1.0), Leu125 (-0.9)
**d2**	Ala120 (-2.3), Lys110 (-1.9)	Leu130 (-6.9), Thr190 (-4.9), Tyr162 (-2.1)
**d3**	Ala113 (-1.5), Ala120 (-1.2), Gly123 (-1.0), Gly93 (-1.5), Lys185 (-1.3), Thr192 (-1.3), Val203 (-3.4)	Ala113 (-4.0), Ala115 (-1.1), Ala117 (-1.0), Arg164 (-1.0), Gly123 (-1.2), Pro165 (-1.4), Val122 (-1.0), Val189 (-2.1)
**d4**	His111 (-0.9), Ala118 (-1.2), Gly126 (-2.1), Val121 (-1.3)	Asn173 (-1.5), Asn174 (-0.9), Asn181 (-2.6), Lys185 (-3.4)
**d5**	Gly124 (-1.3), Leu130 (-3.4), Met129 (-5.0), Pro165 (-11.4)	Ala115 (-2.6), Ala120 (-4.7), Lys110 (-6.1), Met112 (-4.7)

* weighted values computed considering weights of each conformer reported in Table S3

Table S10 PrP-E200K residues at the **d1-d5** interfaces. Residues in either **A** or **B** unit placed within less than 3.0 Å from the interfacing B or A unit, respectively, are reported in columns **A|B** and **A|B**. Ionizable residues or groups, i.e., Asp, Glu, Arg, Lys, His, C-ter and N-ter, in either A or B unit placed within less than 6.0 Å from the interfacing B or A unit, respectively, involved in long-ranged electrostatic contacts, are reported in columns **∓A|B**

model	A|B	A|B	∓A|B
**d1**	105 108 109 112 121 192 197	115-117 119-122 124 126 127 (125 189)	200 | 178<br>204 | 178
**d2**	117 120-124	116 117 124 125 130-133 160 162 186 190	110 | 221
**d3**	112 121-123 125 181 182 184 185 200 203	111-113 121 129 165 166 188 221 224 225 (115)	Nter | 221
**d4**	121	121 122 125 181 182 184 185 203	185 | 178<br>164 | 207
**d5**	116 117 124 125 127 129 130 165-167 186 189 190 221	118-120 122	221 | 110<br>Cter | 110

Note S4 *The mdp file used for NPT simulation with Gromacs*

```
integrator = md
nsteps = 50000000  ; tot 100 ns
dt = 0.002
constraints = all-bonds
nstlist = 10
cutoff-scheme = Verlet
coulombtype = pme
vdw-type = cut-off
nstcalcenergy = -1
rcoulomb = 1.0
fourierspacing = 0.12
ns_type = grid
rlist = 1.2
rvdw = 1.0
Tcoupl = v-rescale
tau_t = 0.1 0.1
tc-grps = protein non-protein
ref_t = 300 300
Pcoupl = Berendsen              ; comment this line in NVT
Pcoupltype = isotropic          ; comment this line in NVT
tau_p = 0.5                     ; comment this line in NVT
compressibility = 4.5e-5        ; comment this line in NVT
ref_p = 1.0                     ; comment this line in NVT
gen_vel = no
gen_temp = 300
gen_seed = -1
nstxout = 500
nstvout = 500
nstfout = 500

nstenergy = 100
energygrps = system
lincs-iter = 2
DispCorr = EnerPres
optimize_fft = yes
```
